# Effect of Medication Therapy Management by Pharmaceutical Care on Blood Pressure and Cardiovascular Risk in Hypertension: A Systematic Review, Meta-Analysis, and Meta-Regression

**DOI:** 10.3390/ph16060845

**Published:** 2023-06-06

**Authors:** Maurilio de Souza Cazarim, Estael Luzia Coelho Cruz-Cazarim, Kathleen Boyd, Olivia Wu, Altacílio Aparecido Nunes

**Affiliations:** 1 Department of Pharmaceutical Sciences, Faculty of Pharmacy, Federal University of Juiz de Fora, Juiz de Fora 36036-900, MG, Brazil; 2 Department of Pharmaceutical Service, Faculty of Pharmaceutical Sciences of Ribeirão Preto, University of São Paulo, Ribeirão Preto 14040-903, SP, Brazil; 3 Health Economics and Health Technology Assessment, University of Glasgow, Glasgow, G12 8QQ, UK; 4 Department of Social Medicine, Ribeirão Preto Medical School, University of São Paulo, Ribeirão Preto 14048-900, SP, Brazil; altacilio@fmrp.usp.br

**Keywords:** hypertension, evidence-based pharmacy practice, primary health care, heart disease risk factors, primary prevention

## Abstract

Medication therapy management by pharmaceutical care (MTM-PC) has been shown to improve the effectiveness of antihypertensive treatments. The aim was to answer the question: what are the MTM-PC models and their impact on hypertensive patients’ outcomes? This is a systematic review with meta-analysis. The search strategies were run on 27 September 2022 in the following databases: PubMed, EMBASE, Scopus, LILACs, Central Cochrane Library, Web of Science; and International Pharmaceutical Abstracts. The quality and bias risk was assessed by the Downs and Black instrument. Forty-one studies met the eligibility criteria and were included, Kappa = 0.86; 95% CI, 0.66–1.0; (*p* < 0.001). Twenty-seven studies (65.9%) had MTM-PC models outlined by the clinical team, showing as characteristics the mean of 10.0 ± 10.7 months of follow-up of hypertensive patients, with 7.7 ± 4.9 consultations. Instruments to assess the quality of life measured the enhancement by 13.4 ± 10.7% (*p* = 0.047). The findings of the meta-analysis show a mean reduction of −7.71 (95% CI, −10.93 to −4.48) and −3.66 (95% CI, −5.51 to −1.80), (*p* < 0.001) in mmHg systolic and diastolic pressures, respectively. Cardiovascular relative risk (RR) over ten years was 0.561 (95% CI, 0.422 to 0.742) and RR = 0.570 (95% CI, 0.431 to 0.750), considering homogeneous studies, I² = 0%. This study shows the prevalence of MTM-PC models outlined by the clinical team, in which there are differences according to the models in reducing blood pressure and cardiovascular risk over ten years with the improvement in quality of life.

## 1. Introduction

About 67% of deaths in the world (38 million) occur due to non-communicable chronic diseases (NCDs). A World Health Organization (WHO) projection presents the number of NCD deaths increasing each year, with an increase of three million more deaths every four years [1,2]. Among the NCDs, cardiovascular diseases (CVDs) stand out, which are responsible for 17.9 million annual deaths worldwide. Systemic arterial hypertension is of great importance among CVDs, as it is highly prevalent in the world population and can be considered an aggravating factor for other CVDs [3,4].

Hypertension affects approximately 45–67% of elderly individuals and 2–12% of children and adolescents worldwide. The prevalence in adult individuals is estimated at approximately 19–42.5% in emerging countries. Its severity is related to the impairment of target organs, such as kidneys, heart, and brain [1,3,5]. According to the WHO, hypertension is considered the main risk factor for the occurrence of other diseases of the circulatory system [2], as it is known that an increase of 10 mmHg in systolic blood pressure is capable of increasing by 25% the risk of developing CVDs, representing a risk association equal to 1.2 in observational studies [6,7].

Health technologies characterized as services capable of managing treatment preventively become contributory to the management of hypertensive patients with the promise of producing results of better effectiveness in blood pressure control and cardiovascular risk reduction [8,9]. Pharmaceutical care (PC) is a model of professional practice that constitutes a set of actions and services performed by the pharmaceutical professional, which considers the biopsychosocial sphere of the individual, family, and community, working along with the health team, focusing on the prevention and resolution of problems of health, further to the promotion, protection, damage prevention, and recovery of health, including not only the clinical assistance dimension but also the technical pedagogical dimension of health work. In this model of practice, the pharmacist assumes responsibility for managing people’s healthcare, which must be shared with the health team and the actions agreed upon with the patient/family [10,11,12].

PC works on adherence to medication, lifestyle changes, dietary sodium restriction, moderation of alcohol consumption, a balanced diet, weight reduction, regular physical activity, and smoking cessation, which are important changeable factors in the management of hypertension. In this way, PC develops a service capable of modifying those factors and providing care that improves the effectiveness and safety of the treatment—medication therapy management (MTM). Thus, MTM-PC is a health technology that is very important in enabling the aid of hypertension management regarding classic treatment with calcium channel blockers (CCB), angiotensin converting enzyme inhibitors (ACE inhibitors or ACE-I), angiotensin receptor blockers (ARBs), and diuretics, and is also related to treatment with new drugs [9,10].

The PC results related to MTM show that there is a positive clinical impact on the reduction in blood pressure and cardiovascular risk, but the impact of different models, as well as their results regarding the scenario in which it is inserted, are unknown in the literature [13,14]. Furthermore, in health management and planning, decision-making based on the highest possible degree of evidence is necessary [15,16,17]. Even if the PC produces good results for the treatment of hypertension, it is necessary to evaluate the existing MTM-PC models, their characteristics and the influence of the scenarios for their insertion in order to define a profile adjustable for each reality that generates results with greater precision, in order to incorporate this service as a feasible health technology for different realities of healthcare systems [8,18].

Decision-making processes in health are carried out either in a subjective way, which is more related to the previous conceptualizations of the decision maker and their subjectivity, or in a systematized way, which is more rational and is revealed to culminate in the most effective process. In the latter, it is essential to observe the sources of information as well as the origins of the results that will support decision-making [19]. The greater the robustness of the analysis on the source of information for decision-making, the greater the assertiveness of the decision. In addition, the highest degree of evidence must be considered. In view of the classifications of epidemiological studies, the satisfactory degree of evidence for assertive decision-making in health is at the top of Chiappelli’s pyramid, such as systematic review studies with meta-analysis. In this sense, a systematic review with meta-analysis and, mainly, meta-regression is capable of assigning better precision in the results to answer the review question and also to distinguish itself from other reviews. There are some reviews on this theme, but they are poor at describing different services and models of MTM-PC, as well as summarizing the characteristics of those models compared to their results [15].

This study was reasoned on the hypothesis that PC is a health technology to be incorporated into health systems to improve effectiveness in reducing blood pressure and cardiovascular risk in hypertensive patients, and its effects may differ and be measured in different models of MTM. In this context, the aim of this study was to generate evidence for the treatment effect on blood pressure and cardiovascular risk from different models of MTM by PC for hypertensive patients in the context of primary healthcare.

## 2. Results

**Figure 1:**
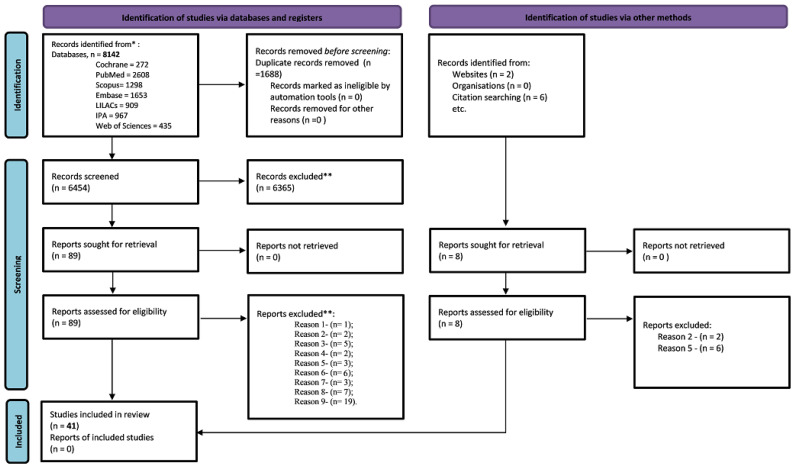
Prisma flowchart for the search strategy and selection performed in the systematic review. Caption: * report of the number of records identified from each database or register searched; ** classification of exclusion for the studies: reason one—non-pharmaceutical interventions; reason two—evaluates the service and not outcomes; reason three—does not assess the outcomes of the review; reason four—the service is not suitable for review/intervention at a tertiary level; reason five—describes the results of the program; reason six—the methodology does not fit the review criteria; reason seven—there is not necessarily contact with the patient; reason eight—it is not necessarily a hypertensive patient; reason nine—clinical protocol, published abstract, case report, and review. Adapted from: Page, M.J.; McKenzie, J.E.; Bossuyt, P.M.; Boutron, I.; Hoffmann, T.C.; Mulrow, C.D.; et al. The PRISMA 2020 statement: an updated guideline for reporting systematic reviews. *BMJ* **2021**, 372, 71. https://doi.org/10.1136/bmj.n71. For more information, available: http://www.prisma-statement.org/; accessed on 23 August 2022.

The initial search yielded 8142 records of which 89 articles underwent full-text evaluation, and 41 articles were selected and 48 were excluded (Table S1). Their selection was consistent with the researchers’ agreement [Kappa = 0.86; 95% CI, 0.66–1.0; (*p* < 0.001)]. It is noteworthy that in the search carried out in the grey literature, no study was found that met the inclusion criteria and mastery of the review after reading the full text (Figure 1). 

According to the 41 studies eligible for this systematic review, it was possible to report that the countries with more published studies within this theme are the USA with 17 (41.5%) and Brazil, 5 (12.2%). Among these studies, 25 (61.0%) are randomized controlled trials (RCTs); 3 (7.3%) are clinical trials without randomization; 10 (24.4%) represent clinical trials defined as quasi-experimental; 3 (7.3%) are observational studies; and 32 studies (78.1%) present sample calculation or at least report the number of patients eligible for the study. The mean duration of the studies is 18 ± 16.5 months and, 24 (58.5%), 9 (21.9%), and 15 (36.6%) studies feature randomization, blinding, and patient allocation concealment, respectively. The total number of patients in the intervention group is 4195 and in the control group 4978, with respective follow-up losses of 1338 and 913 (Table 1).

The quality of the studies was assessed and discriminated by the classification of Downs and Black [61]. The highest score achieved is 96.3 and the lowest is 22.2. The percentage of studies classified as having high evidence is 19.5% and with flawed evidence is 29.3% (Table 2).

In the qualitative evaluation of the MTM models from PC, it was possible to measure the mean of 10.0 ± 10.7 months of follow-up and a median of 6 months, with a minimum and maximum of 2–24 months. The mean of consultations is 7.7 ± 4.9 and the consultation time is 29.0 ± 8.0 min. The clinical scenario consists of 19 (44.2%) services developed in community pharmacies, 17 (39.5%) in healthcare facilities, and 7 (16.3%) in outpatient clinics. The most prevalent MTM models are those based on methods developed by the clinician themselves, an own model, 27 (65.9%); followed by the pharmacotherapy workup method 7 (17.1%); Dáder 5 (12.2%); and SOAP 1 (2.4%). Most MTM models have an educational/patient empowerment character, 34 (82.9%), and are structured with multidisciplinary support, even if it is just a pharmacist/physician, 26 (63.4%) (Table 3).

According to the total of 9173 patients, a profile could be defined whereby the mean age is 61.6 ± 6.6 and 61.9 ± 5.5 years for the intervention and control groups, respectively. In addition, there is a higher prevalence of male patients, with high education, and white skin color for both groups (Table 4).

The number of patients with diabetes; smokers; and alcoholics is992 (30.6%) and 1236 (33.8%); 491 (21.2%) and 473 (22.1%); and 257 (19.5%) and 263 (21.7%) for the intervention and control/exposure groups, respectively. Most patients are level 1 and 2 obese, totaling 1740 (60.0%) in the intervention group, and 1424 (57.2%) in the control group. Among the previous history of diseases associated with hypertension, ischemic heart disease has the highest prevalence, with 227 (17.8%) and 255 (20.1%) patients for the intervention and control groups, respectively. It is noteworthy that out of the nine studies that evaluate the quality of life, 88% of them show that there was an improvement in the quality of life of hypertensive patients followed-up by the MTM-PC, which was measured by standard instruments to assess the quality of life. It is highlighted that the mean increase in the score of quality-of-life was 13.4 ± 10.7% (*p* = 0.047) (Table 5). 

The proportion of pressure control profile is 1673 (38.1%) and 2350 (63.3%) patients at the end of follow-up when compared the group with conventional care and the group with MTM by PC, respectively. The proportion of patients with total cholesterol, HDL, LDL, and triglycerides at satisfactory levels for both groups is 172 (32.7%) and 314 (59.6%) (*p* = 0.001); 200 (37.9%) and 206 (39.2%), (*p* = 0.650); 236 (44.9%) and 341 (64.8%), (*p* = 0.001); and 240 (45.5%) and 290 (55.0%), (*p* = 0.002), respectively (Table 5).

Regardless of the study design, there is a reduction in blood pressure when comparing the control/exposure and intervention groups. However, neither the study by Tobari et al. [59], a randomized clinical trial, or Erickson et al. [54], an observational study, have any evidence of improvement in blood pressure and the study by Robinson et al. [57], a non-randomized clinical trial, shows evidence of difference only for systolic pressure. Mean reductions in systolic (SBP) and diastolic blood pressure (DBP) are 6.8 and 3.7 for randomized clinical trials, 17.3 and 10.2 for non-randomized clinical trials, and 3.9 and 0.6 for observational studies, respectively (Table 6).

**Figure 2:**
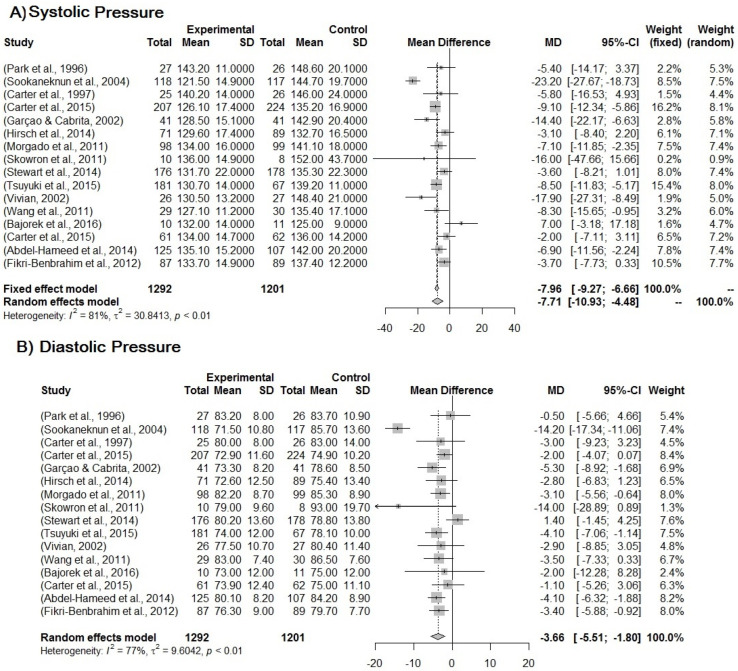
(**A**) Forest plot for systemic blood pressure. (**B**) Forest plot for diastolic blood pressure. Forest plot of blood pressure reduction in the intervention group compared to the control group in relation to MTM by PC for the treatment of hypertension. Included studies: Park et al. [23]; Sookaneknun et al. [24]; Carter et al. [29]; Carter et al. [30]; Garçao, Cabrita [34]; Hirsch et al. [35]; Morgado, Rolo, Castelo-Branco [39]; Skowron, Polak, Brandys [42]; Stewart et al. [43]; Tsuyuki et al. [45]; Vivian [46]; Wang et al. [47]; Bajorek et al. [48]; Carter et al. [49]; Abdel-Hameed et al. [51]; Fikri-Benbrahim et al. [52].

The randomized clinical trial studies with quality assessed as good-to-high evidence were selected to be included in the meta-analysis. The treatment effect of MTM-PC was analyzed firstly for blood pressure. The mean reduction in blood pressure in the intervention group compared to the control group is −7.71 (95% CI, −10.93 to −4.48) and −3.66 (95% CI, −5.51 to −1.80) (*p* < 0.001) for SBP and DBP, respectively. It is important to consider the random model for the results due to the high heterogeneity between the studies, which is 81% and 77% (Figure 2).

**Figure 3:**
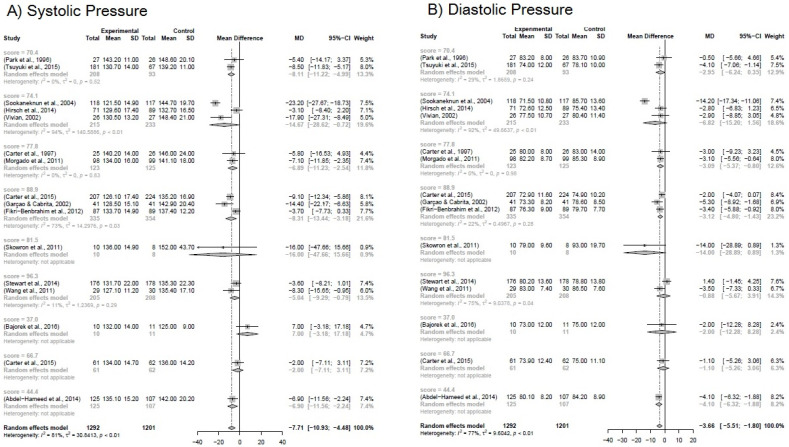
(**A**) Forest plot for systemic blood pressure. (**B**) Forest plot for diastolic blood pressure. Forest plot of blood pressure reduction in the subgroup analysis for the study quality score. Included studies: Park et al. [23]; Tsuyuki et al. [45]; Sookaneknun et al. [24]; Hirsch et al. [35]; Vivian [46]; Carter et al. [29]; Morgado, Rolo, Castelo-Branco [39]; Carter et al. [30]; Garçao, Cabrita [34]; Fikri-Benbrahim et al. [52]; Skowron, Polak, Brandys [42]; Stewart et al. [43]; Wang et al. [47]; Bajorek et al. [48]; Carter et al. [49]; Abdel-Hameed et al. [51].

**Figure 4:**
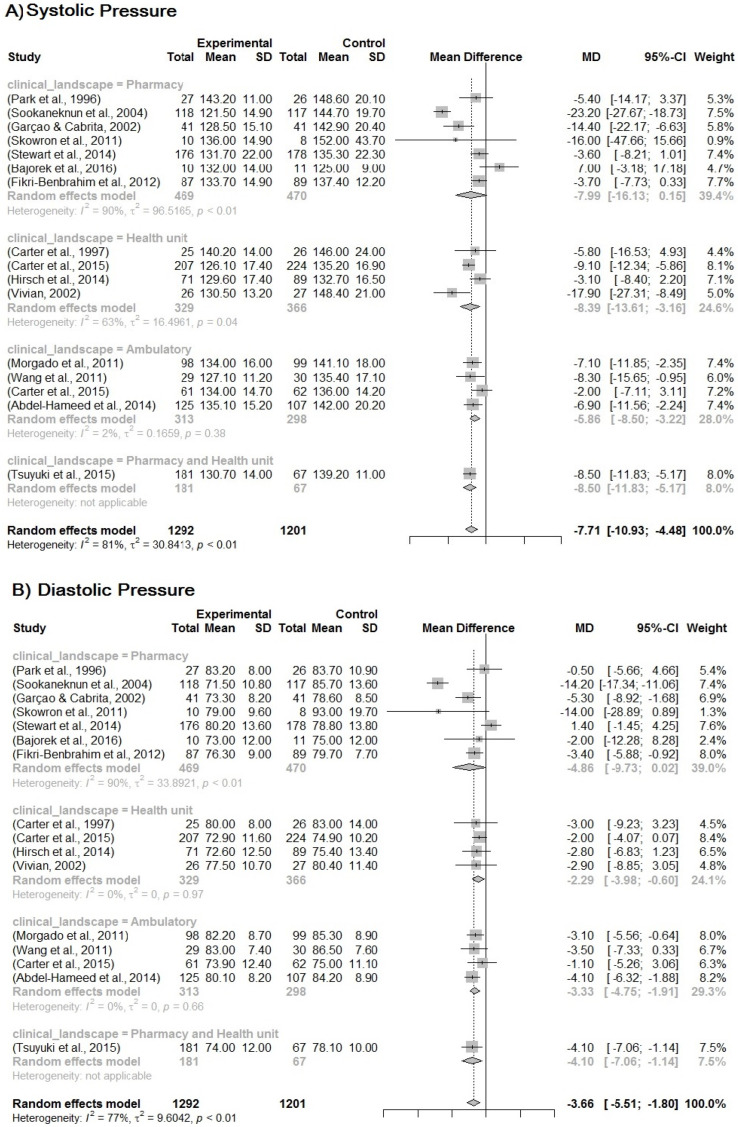
(**A**) Forest plot for systemic blood pressure. (**B**) Forest plot for diastolic blood pressure Forest plot of blood pressure reduction in the subgroup analysis for the MTM-PC insertion scenario. Included studies: Park et al. [23]; Sookaneknun et al. [24]; Garçao, Cabrita [34]; Skowron, Polak, Brandys [42]; Stewart et al. [43]; Bajorek et al. [48]; Fikri-Benbrahim et al. [52]; Carter et al. [29]; Carter et al. [30]; Hirsch et al. [35]; Vivian [46]; Morgado, Rolo, Castelo-Branco [39]; Wang et al. [47]; Carter et al. [49]; Abdel-Hameed et al. [51]; Tsuyuki et al. [45].

**Figure 5:**
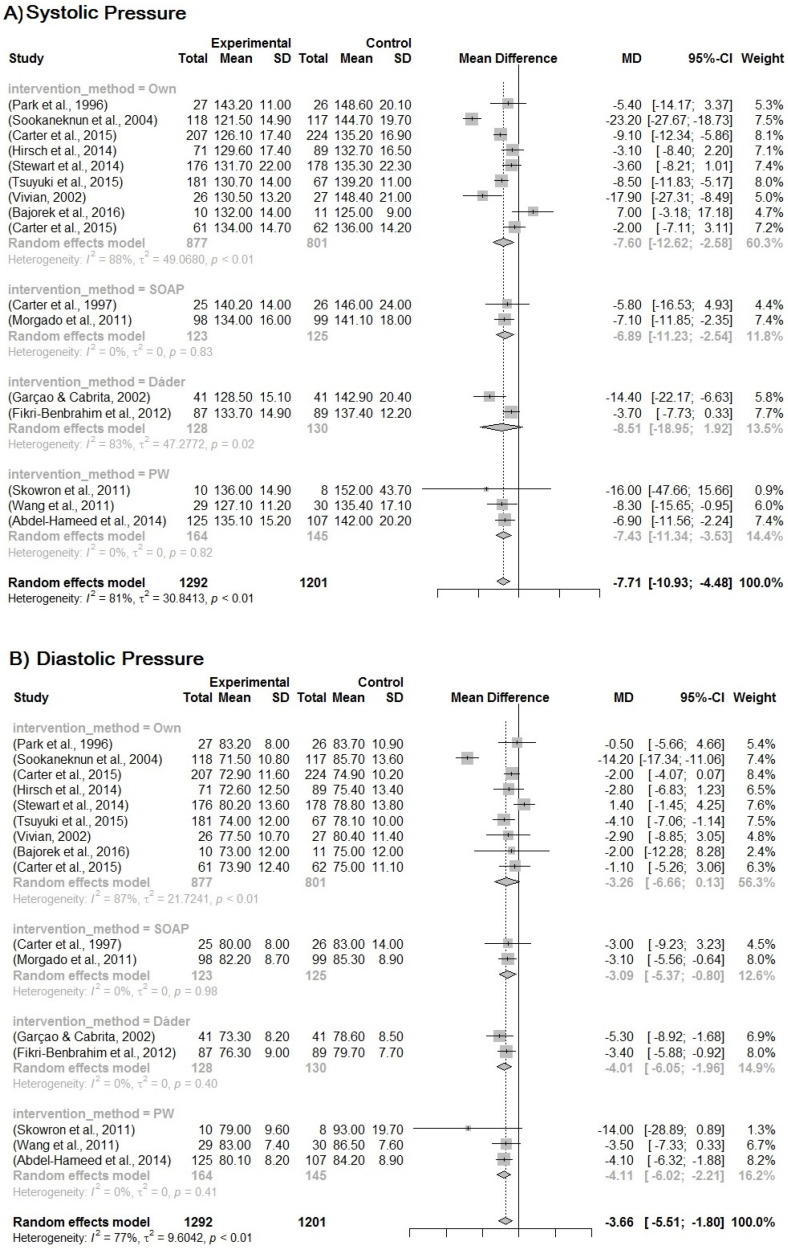
(**A**) Forest plot for systemic blood pressure. (**B**) Forest plot for diastolic blood pressure. Forest plot of blood pressure reduction in the subgroup analysis for the MTM-PC models. Included studies: Park et al. [23]; Sookaneknun et al. [24]; Carter et al. [30]; Hirsch et al. [35]; Stewart et al. [43]; Tsuyuki et al. [45]; Vivian [46]; Bajorek et al. [48]; Carter et al. [49]; Carter et al. [29]; Morgado, Rolo, Castelo-Branco [39]; Garçao, Cabrita [34]; Fikri-Benbrahim et al. [52]; Skowron, Polak, Brandys [42]; Wang et al. [47]; Abdel-Hameed et al. [51].

Due to the heterogeneity between the studies, a sub-analysis was performed based on the group of different variables: study quality score (0–100% according to the level of evidence); clinical setting (where the PC was developed, pharmacy, facilities, clinic); method for developing the MTM (own, Dáder, PW, SOAP). It is likely that if we removed the studies by Vivian et al. [46] and Skowron et al. [42], heterogeneity between studies may decrease, although in this case, the *p*-value is 0.0994. The same situation is represented for the other subgroup analyses, whose *p*-value is 0.6143 and 0.9916, for the clinical scenarios and the method, respectively. The results are shown in Figure 3, Figure 4 and Figure 5. 

**Figure 6:**
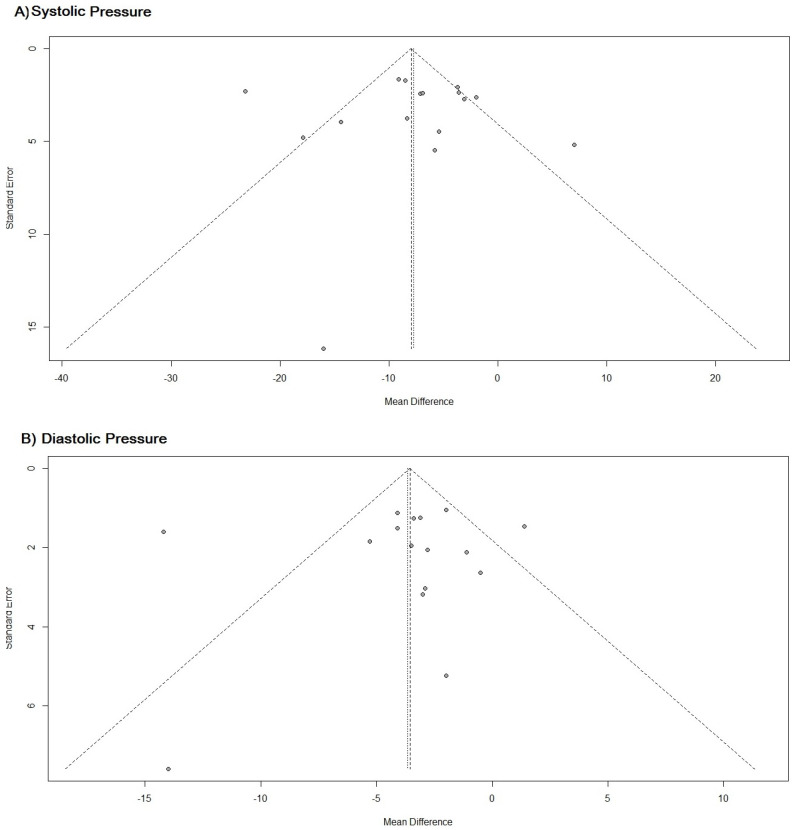
(**A**) Funnel chart for systemic blood pressure. (**B**) Funnel plot for diastolic blood pressure. Funnel plot of studies on the impact of MTM by PC on blood pressure in hypertensive patients.

The funnel graph shows that there is considerable accuracy in the results of the studies and it also shows that there is no publication bias influencing the results, *p*-value = 0.8539 (Figure 6).

**Figure 7:**
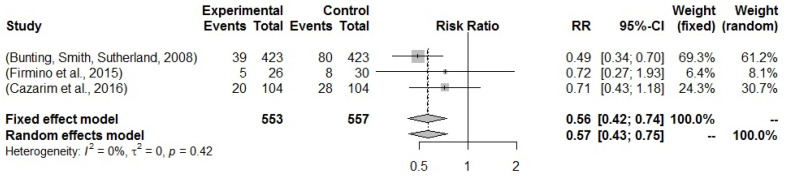
Forest plot of studies for which it possible to assess the relative risk regarding the impact of MTM by PC on cardiovascular risk over ten years in hypertensive patients. Included studies: Bunting, Smith, Sutherland [21]; Firmino et al. [33]; Cazarim et al. [60].

Relative risk was analyzed for cardiovascular risk over ten years, as measured by the ASCVD risk scale. It was only possible to calculate the risk in three studies. The results show that there is a reduction in the cardiovascular risk of hypertensive patients when there is MTM by PC for hypertensive patients. In this sense, the PC works as a protective factor when analyzed both by the fixed model and by the random model, relative risk (RR) = 0.561 (95% CI 0.422–0.742) and RR = 0.570 (95% CI 0.431–0.750). Although the studies can be considered homogeneous, I² = 0%, it makes no sense to state this possibility, due to the smaller number of studies for the calculation of “*n*” in the I² formula (Figure 7).

**Figure 8:**
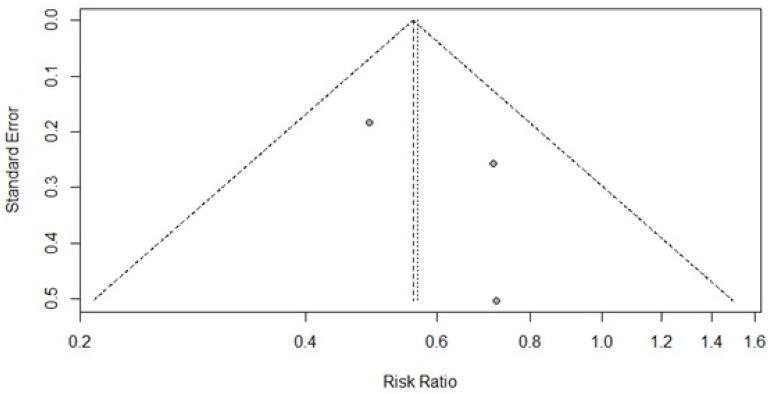
Funnel plot of the studies that made it possible to assess the relative risk regarding the impact of MTM by PC on cardiovascular risk over ten years in hypertensive patients.

The funnel plot for analysis of cardiovascular risk shows that there is a lower possibility of publication bias; although there are few studies for this analysis, the tendency would be to increase the RR. However, there is no asymmetry to support a bias, *p* = 0.4949. It is noteworthy that more than 50% of the studies show good accuracy, greater than 30% (Figure 8).

**Figure 9:**
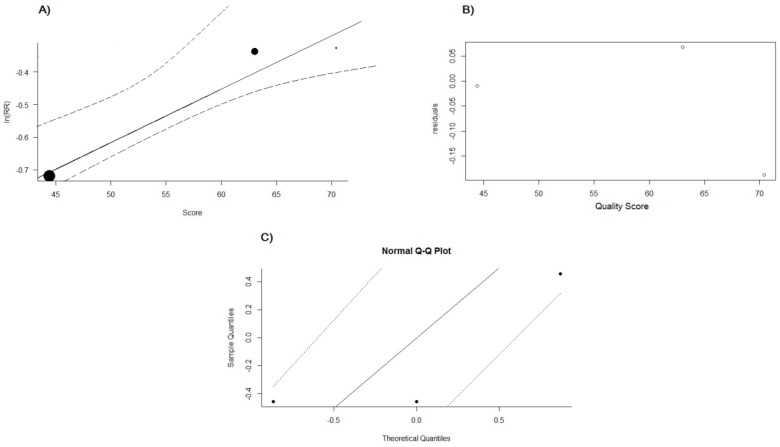
(**A**) Meta-plot for cardiovascular risk and study quality score. (**B**) Meta-regression graph for cardiovascular risk and study quality score. (**C**) Normal Q–Q plot meta-regression for cardiovascular risk and study quality score. Meta-regression for mixed-effects models for cardiovascular risk and the quality score of studies referring to MTM by PC for hypertensive patients.

Data meta-regression was performed, as the quality scores of the studies may be associated with their results regarding cardiovascular risk over ten years. It is not possible to achieve a level of good evidence due to the number of studies, but meta-regression may suggest that the higher the study quality, the lower the protective impact of PC on cardiovascular risk, *p* = 0.2532 (Figure 9).

## 3. Discussion

The selection of studies for the review has a satisfactory agreement between the two researchers, classified as almost perfect, which shows that the review is consistent with its protocol regarding the robustness of the methods used for the inclusion of studies [62]. The quality evaluation of the included studies shows that the clinical trial studies have better scores than the observational studies in general, 70.7%, and the greatest number of studies included in the review have a score above 60 (0–100). The mean quality score is 70.8 ± 19.3 for randomized clinical trials, 52.7 ± 15.3 for non-randomized clinical trials, and 51.8 ± 19.3 for observational studies. However, studies with higher chances of bias, classified as poor or flawed evidence (51.2%), surpass the total number of studies with good and high evidence (48.8%). It is noteworthy that the better the quality of the study, the greater the chances of having better accuracy of the results, and this could be verified in this review, in which the types of studies with a higher level of evidence present positive results from MTM-PC [15,63].

Pharmaceutical interventions in the MTM process, either alone or in collaboration with other health professionals, are recognized to improve blood pressure control. However, pharmaceutical interventions can have a magnitude of differential effects on blood pressure ranging from very large, modest, to no effect. This fact can be determined by the heterogeneity of the MTM-PC models and in the methods of the study that evaluate that model [64]. It is noteworthy that this review shows that most MTM-PC models are developed by the pharmacist or clinical group, the own model grounded at default methods, maybe because they are better adapted to each reality.

Additionally, when analysing those studies that present poor results for blood pressure control compared to the others in this review, we find that there is no interference. In this case, it is noted that the studies by Bajorek et al. [48] and Modé et al. [55] present results in which MTM does not promote improvement in blood pressure values. The quality of studies that show improvement in blood pressure values was compared with the quality of these two studies. The adjusted value of the Mann–Whitney test, w = 7.00 (*p*= 0.036), shows that the quality of these two studies, measured by the Downs and Black [61] instrument, is lower than the other studies that show improvement in arterial blood pressure values.

It is noteworthy that there is evidence for homogeneity between the groups compared in this review regarding sociodemographic characteristics, as well as for the variables that cannot be modified by the intervention of the studies (staging of the degree of hypertension, presence of diabetes, smoking, alcoholism, obesity, body mass index, abdominal circumference, and history of diseases). Homogeneity between groups is an important factor for comparing them and obtaining the measure of the clinical effect of an intervention, which gives greater accuracy to the results of this review [65].

In addition, there is better pressure control of patients in MTM by PC, of which 25.2% more have blood pressure control and improvement in lipid profiles compared to the group with conventional health care. The importance of controlling blood pressure and improving the lipid profile directly infers in the management of hypertensive patients, both clinical regarding the reduction in cardiovascular risk, and managerial for improving the profile care in the sense to pass from emergency to a preventive scope and, consequently, to promote changes in the pharmacotherapy [60].

The meta-analysis of this study shows an RR less than 1 [0.56; 0.42–0.74, 95% CI] for the cardiovascular risk over ten years, which represents a protective character for hypertensives regarding the CP treatment effect [66]. The measure of effect is able to show the efficacy of MTM by PC equal to 44.0% (26.0–58.0, 95% CI), an important result when compared to other preventive interventions [67]. It is like that this result may have a strong association with pharmaceutical interventions related to drug-related problems, which have an impact on the effectiveness and safety of pharmacological treatment, with a consequence of improved adherence and lifestyle [68,69].

In addition, there is evidence for a mean reduction in systolic and diastolic pressures. These results are relevant, as a decrease of 5.7 mmHg is capable of reducing the absolute risk of acute myocardial infarction related to ischemic heart disease, stroke, and heart failure by 3.7 years, by 2.00%, 2.40%, and 2.20%, respectively. This study shows a reduction between 4.48 to 10.93 mmHg in systolic pressure and 1.80 to 5.51 mmHg in diastolic pressure. Consequently, it can impact on the absolute risk reduction over ten years, reaching 8.74% for health complications from hypertension and 1.13% for the reduction of morbidities associated with hypertension [70].

The subgroups’ analysis for quality of the studies, the clinical setting, and the follow-up models contribute to these results. The differences in the clinical scenario show that the community pharmacy presents a higher impact on blood pressure reduction, but these subgroups have the study of Bajorek et al. [48], which presents negative results for blood pressure. It could influence the confidence interval for this group. However, the other scenarios have evidence for reducing blood pressure. Although there are some differences among the models of MTM-PC on blood pressure, all models are shown to be efficient. There is evidence for own models to be able to cause the better mean difference in blood pressure, −7.60 [IC 95%, −12.62; −2.58] and −3.26 [IC 95%, −6.66; −0.13], followed by SOAP −6.89 [IC 95%, −11.23; −2.54] and −3.09 [IC 95%, −5.37; −0.80]. Additionally, the PW model has a good balance for reducing systolic and diastolic pressure, PW −7.43 [IC 95%, −11.34; 3.53] and −4.11 [IC 95%, −6.02; −2.21]. The Dáder model has a confidence interval passing by one, which means that it can have no good results for blood pressure, despite it presenting a better range for reducing blood pressure −8.51 [IC 95%, −18.95; 1.92] and −4.01 [IC 95%, −6.05; −1.96].

The results show that these subgroups do not influence the discrepancies in results between studies for blood pressure control, (*p*-value > 0.05). Thus, it is noted that the hypothesis that the different models of MTM by PC can influence, in a positive or negative result, does not apply [64]. Therefore, it is possible that MTM-PC models, which are not standard to the existing philosophy of PC and that are not adapted to the regional reality, cultural, and epidemiological characteristics, and to the needs of the health systems, are capable of providing discrepant and even ineffective results for reducing arterial blood pressure [71].

The result of the clinical impact of PC can have repercussions on the health system as a whole, since primary health care is recommended to be resolutive and preventive to improve the efficiency of the system. In this sense, it is noteworthy that MTM by PC is able to reduce hospital readmissions of hypertensive patients by an average of 30 days [72]. It is known that as the level of complexity of care increases, the cost per patient for the health system also increases. In this way, PC may be able to optimize resources and save costs in health systems, with the ability to improve the patient’s quality of life, as evidenced in the results of this review [73].

It is noteworthy that the results of this meta-analysis refer to hypertensive patients undergoing preventive care and follow-up in primary health care. Thus, the profile of the MTM models by PC can be delineated with the average number of eight consultations, with an average duration of 30 min among all consultations, with the first consultation taking the longest time from around 40 min to 1 h and 50 min, with a mean of ten months and median of six months of patient follow-up. In a direct cost analysis, it is shown that optimizing resources tends to be more cost-effective in six months of follow-up of patients with MTM by PC [70], which can cost USD 75 to increase in a unit the blood pressure control of hypertensive patients [74]. In the cost-effectiveness analysis of the MTM by PC for hypertensive patients, it is shown that the initial investment in the service is rewarded in outcomes and in return on investment even after three years of patient discharge, presenting the cost of USD 128.03 for improving by one unit the blood pressure of hypertensive patients [75].

In addition, it is highlighted in the profile of the MTM models developed in PC that community pharmacies and primary health units are the most prevalent scenarios for their insertion in the scope of primary health care. Additionally, added to the results of the MTM profile developed in the PC in this review, the important role of health education and the insertion of other PC services in its development in an interdisciplinary and collaborative way with other clinicians is highlighted, such as through pharmacotherapy review, medication reconciliation, therapeutic medication monitoring, and health condition management [71].

This study had some limitations. Several included studies had incomplete data for the cardiovascular risk calculating, and this fact made it difficult to measure the cardiovascular risk for different models and scenarios. If we tried to estimate the cardiovascular risk for different MTM-PC models and scenarios, we would need to perform another review, which certainly would completely change the aim of this review and not evaluate the influence of different models of MTM-PC and other important characteristics of their effects, since there are different instruments to calculate the cardiovascular risk, which must be considered when the calculation is ready in the study, and also there are different diseases that are applied to cardiovascular risk for their management [76,77].

The most important models for MTM-PC in this theme are identified in the included studies, but there are other models such as therapeutic outcomes monitoring (TOM), OLD CARTS, and others that are not identified. Actually, these methods are unhabitual by PC, and they are not very well incorporated into the clinical practice [71]. Consequently, it would not impact on the evidence level of the MTM-PC on hypertension management.

In fact, it is possible to refer to the fact that the MTM-PC can be an adjuvant health technology to new antihypertensive therapies when in the market clinical phase, most likely due to carrying out nonpharmacological lifestyle interventions along with antihypertensive drug therapies [78]. Consequently, it can aid to improve the numbers of poor blood pressure control, which are alarming, as only 10% on average have their blood pressure controlled in low-income and middle-income countries [79].

## 4. Materials and Methods

### 4.1. Review Question

This study is a systematic review with meta-analysis, which set off from the following review question: What MTM models in PC have been developed for hypertensive patients in primary care and what is their clinical impact in blood pressure and cardiovascular risk?

### 4.2. Register

This study had its protocol registered in the PROSPERO database, which makes this paper clearer regarding its production. It can be accessed at https://www.crd.york.ac.uk/prospero/, where ID registration is CRD42017079761. Accessed on 12 December 2022.

The review was designed for the following steps: a question based on the need for evidence; search strategies; deletion of duplicates; primary selection (reading the title and abstract); eligibility; secondary selection (reading the whole text); search in the grey literature, performed by manual search (search in the references of selected articles or indication of experts in the subject) and search in unofficial databases, Google^®^ and clinical trials.gov; data extraction; assessment of the quality of studies; tabulation of results; primary analysis; and performance of meta-analysis. Consensus was established between two blinded researchers in the primary selection stage, and, when necessary, a third researcher intervened to support the inclusion or not of the study according to eligibility and inclusion and exclusion criteria established by the protocol. Eligibility aspects included reliability criteria based on the PRISMA checklist [80].

Search strategies were adjusted in English, Spanish, and Portuguese to four reference databases: PubMed, EMBASE, Scopus, and the Central Cochrane Library. As recommended by the Cochrane manual for systematic reviews, three databases considered local or multidisciplinary/specific to the topic were selected: LILACs, International Pharmaceutical Abstracts (IPA), and Web of Sciences. The descriptors were identified in the scientific dictionaries: Emtree thesaurus, Descriptors in Health Sciences (DeCS), and Medical Subject Headings (MeSH) for their specific databases. The Boolean operator “OR” was used for combining words into the same category and “AND” for combining inter-category [19]. The search was carried out on 27 September 2022. The grey literature search was carried out on 18 November 2022 (Table S2 and Item S1).

The outcomes category, “O” was not considered for this search strategy because it is not a gold standard comparator (placebo or drug, for example) and this could reduce the search sensitivity according to the Cochrane guideline; the outcomes were incorporated with the Boolean operator “OR” in the identification of the study population for the search [80]. A summary of the population (P), interventions (I)/exposition (E), comparators (C), outcomes (O), and study design (S) considered, following the PI(E)COS acronym for the search strategy, with no filters (Table 7).

The studies were selected in the following order: title, abstract, and full-text reading. The selection was carried out using the Rayyan platform for systematic reviews, which is available for registration and, with free access. Data were tabulated in order to extract the general characteristics of the study, sociodemographic characteristics of the patient groups, and clinical and care characteristics, as well as the definitions of the MTM model developed in PC. To access the quality of the studies, the validated instrument by Downs and Black [61] was used, which comprises a checklist of 28 items, which allows for checking the general quality of observational studies and clinical trials, according to the following characteristics: qualities of general aspects of the study, internal and external validity, confounding biases, and the power of the analyses. This measurement is performed by a generated score [61].

The condition or domain of review was defined for the MTM models developed by PC in the context of primary health care, preventive or community care, performed for patients with non-secondary systemic arterial hypertension, and patients treated in the primary care scenario. In addition, it was necessary to judge whether the pharmacist carried out at least two consultations to monitor the patients in at least three months to characterize the clinical follow-up. In addition, it was imperative that the results presented by the study were exclusive to the pharmacist’s intervention, with the pharmacist being incorporated or not in a multidisciplinary team [10,60]. It is important that we have led our analysis to be different in some points of the published reviews on this theme, mainly to describe different services regarding its qualities, as well as the MTM-PC models and the scenarios in which the model is inserted. In this way, we summarize the characteristics of those models compared to their results.

Considerations about the studies eligible for the review were established, such as: considering the original articles to be language-free and considering the studies that sought to answer the question of the systematic review and that met the inclusion and eligibility criteria. It is noteworthy that clinical trials were analyzed in a separate group from other studies and divided into randomized and non-randomized. In addition, meta-analyses and systematic reviews were not included in the review results. Thus, this systematic review considered the following inclusion and exclusion criteria for the studies retrieved in the searches:

Inclusion: studies with adult patients, over 18 years old; outpatients or hypertensive patients seen in community pharmacies or primary/preventive care units; intervention performed based on MTM by PC; minimum of two consultations carried out by the pharmacist in the intervention group; the pharmacist should attend individually or to be a member of a multidisciplinary team, but in this case, the intervention should not depend on the team, but exclusively on the pharmacist; blood pressure assessment should be included as measured results.

Exclusion: studies that considered hypertensive pregnant women; patients with cognitive impairment; patients with moderate to severe chronic kidney disease; patients who were not receiving pharmacological treatment for systemic arterial hypertension; patients without a diagnosis of systemic arterial hypertension; narrative or integrative review studies, dissertations or theses, editorials, news, comments, letters to the editor, abstracts published in the annals of scientific journals or congresses, and guidelines; studies that developed MTM-PC without reviewing pharmacotherapy or pharmacotherapeutic follow-up or did not manage health conditions; studies that did not address MTM in its aspects as a service, on an individual basis, with the elaboration of a therapeutic plan, monitoring the results and with the systematic recording of the patient’s data; studies without a comparator for the results.

### 4.3. Analysis

The analysis of the results was performed using classical statistics. Thus, considering the significance level of 5% and test power equal to 80% [81]. For the classic inferential statistical analysis, MINITAB software version 18 was used. For the systematic review, before the moment of consensus between the two researchers, the agreement between them was analyzed by the Kappa coefficient, with a value above 0.70 being acceptable, otherwise, there would be a need to restructure a new search strategy.

The Mann–Whitney test was performed to assess the difference in quality scores between studies in which the MTM-PC presented favorable clinical results and those with unfavorable clinical results. The score from the Downs and Black instrument [61] was measured in percentages from 0 to 100%. The interpretation of the assessed scores was summarized as follows: up to 50% were considered flawed evidence or irrelevant studies; those between 50–69% were considered poor evidence; between 70–79% were considered good evidence; and 80–100% were considered with high scientific evidence [16,61].

R studio software was used to run the meta-analysis. The effect measure was measured in the meta-analysis by the difference between means for systolic and diastolic blood pressure and by the RR for cardiovascular risk over ten years. The results were diagrammed in a forest plot with the effect measure represented by the diamond. The evaluation of the inconsistency or heterogeneity of the studies was carried out by the I², represented by the percentage of heterogeneity, and analyzed by the *p*-value, which respected the significance level of 5%, thus, the reading of the *p*-value above 0.05 means rejecting the alternative hypothesis and assuming that the studies were homogeneous. However, a homogeneous result may show that there was some random variability in the results of the studies, whereas heterogeneous results represent variability due to inconsistency. The measurement of publication bias in the literature was evaluated using a funnel plot, in which, by diagramming the results, it was possible to verify where there was a tendency for publications for favoring some result. In this case, the better the distribution on the graph and the more homogeneous (symmetric), the smaller the bias [81].

## 5. Conclusions

Most of the studies included in this review have a quality score above 60% and almost half have a good-to-high evidence rating for the results. Among the MTM-PC models analyzed in this systematic review, most are from the USA. Own models, reasoned on standard models, emerge as the most prevalent. Sequentially, from the profile obtained from the MTM by the PC, it was noted that the average time for monitoring hypertensive patients is ten months, with an average of eight 30 min consultations, with the exception of the first consultation being longer, being approximately one hour and 30 min. It is not possible to calculate the average number of patients to be consulted by the pharmacist in the month because many models originate from epidemiological studies and do not refer to the feasibility regarding the capacity of consultation.

There is evidence for the mean reduction in blood pressure and also for better blood pressure control, consequently, there is a reduction in cardiovascular risk over ten years associated with the improvement in quality of life of hypertensive patients assisted in the MTM by PC, which can work as a protective factor to hypertension, presenting a good efficiency to avoid incidence of CVDs in hypertensive patients. Thus, the community pharmacy setting is important for the better reach of MTM-PC impact, but the ambulatory setting has better evidence for reducing blood pressure. Regarding different models, the own model of MTM-PC has the better impact and PW is the most balanced for reducing blood pressure. However, it is highlighted that further exploration is needed.

## Figures and Tables

**Table 1:** Identification and characteristics of the studies selected in the systematic review.

Reference	Year	Country	Study Design	Sampling Planning/Eligible Patients	Study Time (Months)	Randomization	Blinding	Blind Allocation	*N* Intervention Group	*N* Control Group	Losses in Intervention Group	Losses in Control Group
Jamieson et al. [20]	2010	United Kingdom	RCT (open, cross-over, randomized)	81	14	Yes (cross-over, six months)	Yes (patient)	Yes	16	17	0	0
Bunting, Smith, Sutherland [21]	2008	USA	CT (open, cross-over, randomized)	NE	72	No	No	No	423	423	0	0
Erhun, Agbani, Bolaji [22]	2005	Nigeria	CT (Quasi-experimental, single arm)	NE	12	No	No	No	37	37	14	14
Park et al. [23]	1996	USA	RCT	50	7	Yes	No	No	27	26	5	6
Sookaneknun et al. [24]	2004	Thailand	RCT (pre and post-test, randomized)	248	6	Yes	No	No	118	117	5	3
Aguiar et al. [25]	2012	Brazil	CT (quasi-experimental, single arm)	NE	10	No	No	No	35	35	16	16
Aguwa, Ukwe, Ekwunife [26]	2008	Nigeria	CT (quasi-experimental, single arm)	NE	10	No	No	No	24	24	16	16
Bex et al. [27]	2011	USA	Observational (descriptive, before and after)	NE	18	No	No	No	433	433	140	140
Borenstein et al. [28]	2003	USA	RCT	1272	36	Yes	No	No	98	99	206	92
Carter et al. [29]	1997	USA	RCT	50	12	Yes	Yes (investigator -randomization)	Yes	25	26	4	0
Carter et al. [30]	2015	USA	RCT (Cluster)	648	24	Yes	Yes	Yes	207	224	34	30
Chabot et al. [31]	2003	Canada	CT	100	9	No	Yes	No	41	59	7	4
Junior et al. [32]	2008	Brazil	CT (Quasi-experimental, single arm)	NE (convenience sampling)	12	No	No	No	30	30	0	0
Firmino et al. [33]	2015	Brazil	RCT	NE (convenience sampling)	12	Yes	No	Yes	26	30	8	4
Garçao, Cabrita [34]	2002	Portugal	RCT	66	8	Yes	Yes (patient)	Yes	41	41	9	9
Hirsch et al [35]	2014	USA	RCT (pragmatic)	85	24	Yes	No	Yes	71	89	4	2
Hussain et al. [36]	2016	USA	CT (Quasi-experimental, real life; treated and not-treated)	3964	9	No	No	No	229	330	332	0
Kicklighter et al. [37]	2006	USA	CT	NE	8	No	No	No	113	111	0	25
Ortellado et al. [38]	2007	Paraguay	RCT	NE	NE	Yes	No	No	33	28	2	7
Morgado, Rolo, Castelo-Branco [39]	2011	Portugal	RCT	180	12	Yes	Yes (clinical professional)	Yes	98	99	0	0
Parker et al. [40]	2014	USA	CT (quasi-experimental; single arm)	249	30	No (as the aim)	No	No	127	127	122	122
Reid, Murray, Storrie [41]	2005	United Kingdom	RCT	532	9	Yes	No	Yes	92	68	50	32
Skowron, Polak, Brandys [42]	2011	Poland	RCT (intention-to-treat)	193	14	Yes	No	Yes	10	8	18	48
Stewart et al. [43]	2014	Australia	ECR (cluster)	364	6	Yes	No	Yes	176	178	31	10
Taylor et al. [44]	2013	USA	CT (quasi-experimental; single arm)	NE	6	No	No	No	8	8	17	17
Tsuyuki et al. [45]	2015	Canada	RCT	340	49	Yes	No	No	181	67	26	6
Vivian [46]	2002	USA	RCT	53	7	Yes	No	No	26	27	0	0
Wang et al. [47]	2011	China	RCT	60	NE	Yes	No	Yes	29	30	1	0
Bajorek et al. [48]	2016	Australia	RCT	302 (convenience sampling)	18	Yes	No	No	10	11	6	7
Carter et al. [49]	2015	USA	RCT (intention-to-treat)	249	30	Yes	No	Yes	61	62	2	2
Zillich et al. [50]	2015	USA	Observational (case–control)	13,099	12	No	No	Yes	465	1268	0	0
Abdel-Hameed et al. [51]	2014	Egypt	RCT	280	7	Yes	Yes (clinical professional)	No	125	107	15	33
Fikri-Benbrahim et al. [52]	2012	Spain	RCT	116	NE	No (fail)	No	Yes	87	89	2	2
Oparah et al. [53]	2006	Nigeria	CT (quasi-experimental; single arm)	46	NE	No	No	No	36	36	6	NA
Erickson et al. [54]	1997	USA	Observational (prospective Cohort)	128	NE	No	No	NE	40	40	0	0
Modé et al. [55]	2015	Brazil	RCT	NE	5	No	No	NE	10	10	0	0
Lee et al. [56]	2013	Japan	RCT	64	32	Yes	No	No	97	97	1	0
Robinson et al. [57]	2010	USA	CT	376	12	Yes	No	NE	180	196	102	134
Hunt et al. [58]	2008	USA	RCT	302	12	Yes	Yes (clinical professional)	NE	142	103	88	130
Tobari et al. [59]	2010	Japan	RCT	128	12	Yes	Yes	Yes	64	64	2	2
Cazarim et al. [60]	2016	Brazil	EC (quasi-experimental, single arm)	33	72	No	No	No	104	104	47	NA

Caption: CT = clinical trial; RCT = randomized controlled trial; NE = not specified; NA = not applicable.

**Table 2:** Quality assessment of included studies in the systematic review.

Study	Quality Score	Percentage (%)	Mean (SD)	Classification	Relative Frequency
Stewart et al. [43]	26	96.3	89.4 (5.0)	High evidence	19.5%
Wang et al. [47]	26	96.3			
Carter et al. [30]	24	88.9			
Garçao, Cabrita [34]	24	88.9			
Fikri-Benbrahim et al. [52]	24	88.9			
Tobari et al. [59]	24	88.9			
Jamieson et al. [20]	23	85.2			
Skowron, Polak, Brandys [42]	22	81.5			
Carter et al. [29]	21	77.8	73.1 (2.8)	Good evidence	29.3%
Morgado, Rolo, Castelo-Branco [39]	21	77.8			
Sookaneknun et al. [24]	20	74.1			
Hirsch et al. [35]	20	74.1			
Parker et al. [40]	20	74.1			
Vivian [46]	20	74.1			
Robinson et al. [57]	20	74.1			
Park et al. [23]	19	70.4			
Borenstein et al. [28]	19	70.4			
Firmino et al. [33]	19	70.4			
Reid, Murray, Storrie [41]	19	70.4			
Tsuyuki et al. [45]	19	70.4			
Chabot et al. [31]	18	66.7	63.4 (3.9)	Poor evidence	21.9%
Hussain et al. [36]	18	66.7			
Carter et al. [49]	18	66.7			
Hunt et al. [58]	18	66.7			
Zillich et al. [50]	17	63.0			
Erickson et al. [54]	17	63.0			
Cazarim et al. [60]	17	63.0			
Kicklighter et al. [37]	16	59.3			
Lee et al. [56]	15	55.6			
Oparah et al. [53]	13	48.1	37.3 (7.8)	Flawed	29.3%
Bunting, Smith, Sutherland [21]	12	44.4			
Aguwa, Ukwe, Ekwunife [26]	12	44.4			
Abdel-Hameed et al. [51]	12	44.4			
Aguiar et al. [25]	11	40.7			
Junior et al. [32]	11	40.7			
Bajorek et al. [48]	10	37.0			
Erhun, Agbani, Bolaji [22]	9	33.3			
Ortellado et al. [38]	9	33.3			
Bex et al. [27]	8	29.6			
Taylor et al. [44]	8	29.6			
Modé et al. [55]	6	22.2			

Caption: SD = standard deviation. The scores provided by the Downs and Black [61] questionnaire are measured in percentages, which correspond to the 28 items to be answered, with 28 points representing 100%.

**Table 3:** Evaluation of medication therapy management models by pharmaceutical care for hypertensive patients.

Reference	Intervention Time	Consultations Number	Mean-Time of Consultations (Min-Max)	Clinical Scenario	Health Education/Empowerment Intervention	Multidisciplinary Support (Pharmacist/Physician)	Medication Therapy Management Model	Instrument for Measuring Blood Pressure
Jamieson et al. [20]	12 months	12	40 min (NE)	Community pharmacy	No	No	Own (reasoned on PW)	Sphygmomanometer (analog)
Bunting, Smith, Sutherland [21]	72 months	24	30 min (NE)	Community pharmacy	No	No	Own (NE)	NE
Erhun, Agbani, Bolaji [22]	12 months	6	30 min (NE)	Outpatient clinics	Yes	Yes	Own (NE)	NE
Park et al. [23]	6 months	4	25 min (20–30)	Community pharmacy	No	Yes	Own (NE)	Sphygmomanometer (analog)
Sookaneknun et al. [24]	6 months	6	35 min (30–50)	Community pharmacy	Yes	No	Own (NE)	Sphygmomanometer (analog)
Aguiar et al. [25]	10 months	10	45 min (40–60)	Community pharmacy	Yes	No	PW	Sphygmomanometer (analog)
Aguwa, Ukwe, Ekwunife [26]	15 months	5	NE	Community pharmacy	No	No	Own (NE)	NE
Bex et al. [27]	12 months	12	22 min (15–30)	Healthcare facility	Yes	Yes	Own (NE)	NE
Borenstein et al. [28]	12 months	12 or 24	30 min (NE)	Healthcare facility	Yes	Yes	Own (NE)	Digital cuff and mercury sphygmomanometer
Carter et al. [29]	6 months	6	NE	Healthcare facility	Yes	Yes	SOAP	Sphygmomanometer (analog)
Carter et al. [30]	24 months	9 to 24	NE	Healthcare facility	Yes	Yes	Own (NE)	Sphygmomanometer (analog)
Chabot et al. [31]	6 months	3	NE	Community pharmacy	Yes	No	Own (PRECEDE–PROCEED)	Sphygmomanometer (analog)
Junior et al. [32]	12 months	12	40 min (NE)	Healthcare facility	Yes	Yes	PW	Sphygmomanometer (analog)
Firmino et al. [33]	9 months	8	30 min (20–45)	Healthcare facility	Yes	Yes	Dáder	NE
Garçao, Cabrita [34]	6 months	6	NE	Community pharmacy	Yes	No	Dáder	Sphygmomanometer (analog)
Hirsch et al. [35]	9 months	4	30 min (NE)	Healthcare facility	Yes	Yes	Own (PharmD-PCP)	Sphygmomanometer (analog)
Hussain et al. [36]	3 months	3	40 min (NE)	Healthcare facility	Yes	Yes	Own (ReDCHiP CM program)	Sphygmomanometer (analog)
Kicklighter et al. [37]	6 months	6	NE	Community pharmacy	Yes	Yes	Own (NE)	Blood pressure monitors (LifeSource Model 3UA702-V)
Ortellado et al. [38]	6 months	6	NE	Community pharmacy	Yes	Yes	PW	Sphygmomanometer (analog)
Morgado, Rolo, Castelo-Branco [39]	9 months	9	25 min (20–30)	Outpatient clinics	Yes	Yes	Own (bases SOAP)	NE
Parker et al. [40]	6 months	4	NE	Healthcare facility	Yes	Yes	Own (NE)	Digital (HEM 907-XL; Omron; Corporation, Schaumburg, IL, USA)
Reid, Murray, Storrie [41]	5 months	2 to 10	NE	Healthcare facility	No	Yes	Own (NE)	NE
Skowron, Polak, Brandys [42]	13 months	12	NE	Community pharmacy	Yes	Yes	PW	Sphygmomanometer (analog)
Stewart et al. [43]	6 months	6	NE	Community pharmacy	No	No	Own (NE)	Digital (Omron HEM-790IT; Omron Healthcare Co Ltd., Muko, Kyoto, Japan)
Taylor et al. [44]	3 months	3	17 min (15–20)	Community pharmacy	Yes	No	PW	Sphygmomanometer (analog)
Tsuyuki et al. [45]	6 months	6	NE	Community pharmacy; healthcare facility; outpatient clinics	Yes	No	Own (NE)	Digital (BpTRU Medical Devices, Coquitlam, BC, Canada)
Vivian [46]	6 months	6	35 min (30–45)	Healthcare facility	Yes	No	Own (NE)	Sphygmomanometer (analog)
Wang et al. [47]	12 months	12	NE	Outpatient clinics	Yes	Yes	Own (bases PW)	Digital (MOBIL-O-GRAPH^®^,I.E.M GmbH, Cockerillstr.69 D—52222 Stolber Germany)
Bajorek et al. [48]	12 months	12	25 min (15–40)	Community pharmacy	Yes	no	Own (health collaboration model—HCM)	NE
Carter et al. [49]	6 months	4	NE	Outpatient clinics	Yes	Yes	Own (NE)	Digital (HEM 907-XL; Omron, Schaumburg, IL, USA)
Zillich et al. [50]	12 months	12	22 min (15–30)	Healthcare facility	Yes	Yes	Own (NE)	NE
Abdel-Hameed et al. [51]	3 months	3	35 min (20–30)	Outpatient clinics	Yes	Yes	PW	Sphygmomanometer (analog)
Fikri-Benbrahim et al. [52]	5 months	3	NE	Community pharmacy	Yes	Yes	Dáder	Digital (Visomat Comfort 20/40 monitor).
Oparah et al. [53]	6 months	6	NE	Community pharmacy	Yes	Yes	Dáder	Sphygmomanometer (analog)
Erickson et al. [54]	5 months	5	15 min (NE)	Outpatient clinics	Yes	Yes	Own (reasoned on PW)	NE
Modé et al. [55]	4 months	4	NE	Community pharmacy	Yes	Yes	Dáder	Sphygmomanometer (analog)
Lee et al. [56]	6 months	5	24 min (NE)	Healthcare facility	Yes	No	Own (pharmacy outreach service—POS)	Digital (Omron HEM-7011)
Robinson et al. [57]	12 months	3	NE	Community Pharmacy	No	No	Own (pharmaceutical care practice plan)	Digital (Vita Stat BP machine)
Hunt et al. [58]	12 months	4	NE	Healthcare facility	Yes	Yes	NE	Sphygmomanometer (analog)
Tobari et al. [59]	6 months	4	22 min (15–30)	Healthcare facility	Yes	Yes	Own (NE)	Sphygmomanometer (analog)
Cazarim et al. [60]	12 months	12	30 min (20–60)	Healthcare facility	Yes	No	PW	Sphygmomanometer (analog)

Caption: NS = not specified; NA = not applicable, SOAP = subjective, objective, assessment plan; PW = pharmacotherapy workup; Healthcare facility = basic health unit or primary health care unit.

**Table 4:** Results of sociodemographic variables related to patients from studies included in the systematic review.

Variables	Intervention Group (*n* = 4195)	Control Group (*n* = 4978)	*p*-Value
Age mean (SD)	61.6 ± 6.6	61.9 ± 5.5	*p* > 0.05 ^Ψ^
Sex (%)			*p* > 0.05 ^Ψ^
Men	2340 (57.9)	3053 (65.4)	
Women	1700 (42.1)	1611 (34.6)	
Average annual income (%)			*p* > 0.05 ^ⱡ^
>USD 22.000	272 (50.2)	271 (49.8)	
<USD 22.000	207 (48.2)	222 (51.8)	
Education degree			*p* > 0.05 ^ⱡ^
High	600 (38.8)	582 (41.1)	
Middle	384 (24.8)	375 (26.5)	
Low	564 (36.4)	458 (32.4)	
Skin color			*p* > 0.05 ^ⱡ^
White	1303 (54.6)	1611 (49.9)	
Black	589 (24.7)	740 (22.9)	
Other	496 (20.7)	877 (27.2)	

Caption: ^Ψ^ Statistic used to test the homogeneity between groups was the *t*-student test for comparison between means; ^ⱡ^ Statistic used to verify the homogeneity between groups was the chi-square. The value of USD 22,000 was stipulated as the threshold for the salary income of the patients, since it represents 3× the value of the Brazilian gross domestic product in the year 2021, the threshold used in cost-effectiveness studies (SOURCE: worldbank data: Available: https://datatopics.worldbank.org/world-development-indicators/ accessed on 12 October 2022).

**Table 5:** Clinical variables analysis comparing intervention and control/exposure groups from studies included in the systematic review.

Clinical Variables	Intervention Group (*n* = 4195)	Control Group (*n* = 4978)	*p*-Value
Patients Profile			
Staging of the degree of hypertension for inclusion in the study			
Level 1 and 2	829 (19.9)	815 (17.2)	*p* = 1.000
Level 1, 2, 3	3336 (80.1)	3926 (82.8)	
Diabetes (%)	992 (30.6)	1236 (33.8)	*p* = 1.000
Smoking (%)	491 (21.2)	473 (22.1)	*p* = 0.998
Alcoholism (%)	257 (19.5)	263 (21.7)	*p* = 0.999
Obesity			
Normal (%)	189 (6.5)	107 (4.3)	*p* = 0.982
Pre-obesity (%)	970 (33.5)	957 (38.5)	*p* = 0.933
Obesity level 1 and 2 (%)	1740 (60.0)	1424 (57.2)	*p* = 0.992
Body mass index (BMI)	29.2 ± 3.1	29.3 ± 2.3	*p* = 0.760
Abdominal circumference (AC)			
Satisfactory	20 (6.6)	20 (9.4)	*p* = 0.655
Non-satisfactory	306 (93.4)	192 (90.6)	
Prior history of illnesses (%)			
IHD	227 (17.8)	255 (20.1)	*p* = 0.982
ST	63 (4.9)	78 (6.1)	*p* = 0.983
PAD	39 (3.3)	32 (3.3)	*p* = 0.997
HF	43 (3.3)	44 (3.5)	*p* = 1.000
CKD	97 (5.6)	98 (3.9)	*p* = 0.972
Endpoint Analysis			
Medication consumption ± SD ^Ψ^	2.2 ± 0.7	2.0 ± 0.5	*p* = 0.078
Quality of life (% improvement)	13.4 ± 10.7		*p* = 0.047 *
Pressure control (%)	2350 (63.3)	1673 (38.1)	*p* < 0.001 *
Lipemic levels ± SD ^Ψ^			
Total cholesterol	193.4 ± 8.2	214.4 ± 5.8	*p* = 0.057
HDL	48.9 ± 6.9	48.2 ± 7.8	*p* = 0.451
LDL	108.8 ± 13.4	120.6 ± 16.9	*p* = 0.155
TG	168.6 ± 31.2	203.7 ± 40.7	*p* = 0.033 *

Caption: ST = stroke; PAD = peripheral arterial disease; IHD = ischemic heart disease; CKD = chronic kidney disease, SD = standard deviation; HF = heart failure; HDL = high density of lipoprotein; LDL = low density of lipoprotein; TG = triglycerides. Obesity was measured by body mass index according to the 2010 Brazilian obesity guideline when not classified by the study. The percentages in parentheses represent the proportion within the sample number for a given variable, as not all studies in the review consider all these variables. Chi-square with McNemar test was used to test the homogeneity between groups regarding clinical variables. For the quality of life, most studies brought the difference between the quality of life scores; it was not possible to apply a statistical test to verify the difference between the intervention and control groups. ^Ψ^ = Statistical test was applied for continuous variables, with the difference between means evaluated by student-t for paired measures, repeating the significance level of 5%. * = Statistically significant.

**Table 6:** Evaluation of blood pressure values achieved from medication therapy management by pharmaceutical care for different types of studies.

Reference	Study Design	Quality Score	Baseline SBP Intervention Group (after/without Exposition)	Endpoint SBP Intervention Group (after/without Exposition)	SBP Difference Intervention	Baseline DBP Intervention Group (after/without Exposition)	Endpoint DBP Intervention Group (after/without Exposition)	DBP Difference Intervention	Baseline SBP Control Group (before/with Exposition)	Endpoint SBP Control Group (before/with Exposition)	SBP Difference Control	Baseline DBP Control Group (before/with Exposition)	Endpoint DBP Control Group (before/with Exposition)	DBP Difference Control
Randomized controlled trial														
Jamieson et al. [20]	RCT (open, cross-over, randomized)	85.2	142.1 ± NE	129.2 ± NE	12.9	88.0 ± NE	78.8 ± NE	9.2	139.3 ± NE	140.7 ± NE *	−1.4	84.4 ± NE	87.4 ± NE *	−3
Park et al. [23]	RCT	70.4	155.5 ± 21.1	143.2 ± 11.5	12.3	87.8 ± 9.9	83.2 ± 8.0	4.6	147.9 ± 19.6	148.6 ± 20.1 *	−0.7	83.3 ± 8.5	83.7 ± 10.9 *	−0.4
Sookaneknun et al. [24]	RCT (pre and post-test, randomized)	74.1	155.2 ± 15.51	121.5 ± 14.9	33.7	85.7 ± 13.6	71.5 ± 10.8	14.2	142.4 ± 19.8	144.7 ± 19.7 *	−2.3	85.9 ± 12.9	85.7 ± 13.6	0.2
Borenstein et al. [28]	RCT	70.4	162.0 ± NE	140.0 ± NE	22	92.0 ± NE	85.0 ± NE	7	156.0 ± NE	145.0 ± NE	11	90.0 ± NE	84.0 ± NE	6
Carter et al. [29]	RCT	77.8	151.2 ± 21.0	140.2 ± 14.0	11	82.0 ± 9.0	80.0 ± 8.0	2	145.0 ± 19.0	146.0 ± 24.0 *	−1	80.0 ± 9.0	83.0 ± 14.0 *	−3
Carter et al. [30]	RCT (cluster)	88.9	149.8 ± 15.6	126.1 ± 17.4	23.7	86.6 ± 11.6	72.9 ± 11.6	13.7	149.6 ± 15.3	135.2 ± 16.9	14.4	84.3 ± 12.6	74.9 ± 10.2	9.4
Firmino et al. [33]	RCT	70.4	137.7 ± NE	131.5 ± NE	6.2	81.9 ± NE	79.2 ± NE	2.7	132.0 ± NE	136.3 ± NE *	−4.3	79.7 ± NE	83.7 ± NE *	−4
Garçao, Cabrita [34]	RCT	88.9	151.7 ± 23.2	128.5 ± 15.1	23.2	85.7 ± 13.2	73.3 ± 8.2	12.4	147.7 ± 15.9	142.9 ± 20.4	4.8	83.9 ± 9.2	78.6 ± 8.5	5.3
Hirsch et al. [35]	RCT (pragmatic)	74.1	134.8 ± 17.4	129.6 ± 17.4	5.2	75.1 ± 12.5	72.6 ± 12.5	2.5	134.4 ± 16.5	132.7 ± 16.5	1.7	75.7 ± 13.4	75.4 ± 13.4	0.3
Ortellado et al. [38]	RCT	33.3	NE	128.0 ± NE	NE	NE	83.0 ± NE	NE	NE	154.0 ± NE	NE	NE	90.0 ± NE	NE
Morgado, Rolo, Castelo-Branco [39]	RCT	77.8	141.6 ± 16.3	134.0 ± 16.0	7.6	85.2 ± 10.2	82.2 ± 8.7	3	141.9 ± 16.8	141.1 ± 18.0	0.8	86.4 ± 11.7	85.3 ± 8.9	1.1
Reid, Murray, Storrie [41]	RCT	70.4	147.0 ± 17.6	NE	NE	80.0 ± 10.2	NE	NE	145.0 ± 17.6	NE	NE	78.0 ± 10.5	NE	NE
Skowron, Polak, Brandys [42]	RCT (intention-to-treat)	81.5	143.0 ± 12.4	136.0 ± 14.9	7	83.5 ± 10.9	79.0 ± 9.6	4.5	139.5 ± 21.7	152.0 ± 43.7 *	−12.5	90.0 ± 11.9	93.0 ± 19.7 *	−3
Stewart et al. [43]	RCT (cluster)	96.3	141.9 ± 22.4	131.7 ± 22.0	10.2	84.3 ± 14.4	80.2 ± 13.6	4.1	140.1 ± 22.5	135.3 ± 22.3	4.8	83.2 ± 14.5	78.8 ± 13.8	4.5
Tsuyuki et al. [45]	RCT	70.4	149.0 ± 14.0	130.7 ± 14.0	18.3	84.0 ± 12.0	74.0 ± 12.0	10	151.0 ± 11.0	139.2 ± 11.0	11.8	83.0 ± 10.0	78.1 ± 10.0	4.9
Vivian [46]	RCT	74.1	149.0 ± 15.3	130.5 ± 13.2	18.5	89.8 ± 10.9	77.5 ± 10.7	12.3	152.8 ± 14.3	148.4 ± 21.0	4.4	77.9 ± 11.9	80.4 ± 11.4 *	−2.5
Wang et al. [47]	RCT	96.3	136.9 ± 14.9	127.1 ± 11.2	9.8	88.9 ± 9.3	83.0 ± 7.4	5.9	136.5 ± 15.2	135.4 ± 17.1	1.1	88.2 ± 11.5	86.5 ± 7.6	1.7
Bajorek et al. [48]	RCT	37.0	153.0 ± 12.0	132.0 ± 14.0	21	85.0 ± 11.0	73.0 ± 12.0	12	147.0 ± 9.0	125.0 ± 9.0	22	85.0 ± 12.0	75.0 ± 12.0	10
Carter et al. [49]	RCT (intention-to-treat)	66.7	147.0 ± 13.7	134 ± 14.7	13	79.9 ± 11.8	73.9 ± 12.4	6	147.0 ± 11.7	136.0 ± 14.2	11	78.8 ± 9.9	75.0 ± 11.1	3.8
Abdel-Hameed et al. [51]	RCT	44.4	143.0 ± 16.4	135.1 ± 15.2	7.9	85.5 ± 7.3	80.1 ± 8.2	5.4	144.0 ± 20.5	142.0 ± 20.2	2	85.6 ± 9.0	84.2 ± 8.9	1.4
Fikri-Benbrahim et al. [52]	RCT	88.9	140.5 ± 16.1	133.7 ± 14.9	6.8	78.4 ± 9.1	76.3 ± 9.0	2.1	139.5 ± 15.1	137.4 ± 12.2	2.1	79.6 ± 9.2	79.7 ± 7.7 *	−0.1
Modé et al. [55]	RCT	22.2	152.0 ± NE	135.0 ± NE	17	85.0 ± NE	77.0 ± NE	8	128.0 ± NE	127.0 ± NE	1	79.0 ± NE	76.0 ± NE	3
Lee et al. [56]	RCT	55.6	NA	147.04 ± 20.72	NE	NE	71.0 ± 10.97	NE	NE	152.4 ± 18.8	NE	NA	73.8 ± 11.4	NE
Hunt et al. [58]	RCT	66.7	173.0 ± NE	137.0 ± NE	36	90.0 ± NE	75.0 ± NE	15	174.0 ± NE	143.0 ± NE	31	92.0 ± NE	78.0 ± NE	14
Tobari et al. [59]	RCT	88.9	136.5 ± NE	133.6 ± NE	2.9	82.0 ± NE	79.2 ± NE	2.8	137.0 ± NE	135.9 ± NE	1.1	83.5 ± NE	81.2 ± NE	2.3
Mean ± SD			147.5 ± 8.9	132.7 ± 4.9	(14.8 ± 8.9)	84.6 ± 4.0	77.5 ± 4.1	(7.2 ± 4.4)	144.2 ± 9.6	139.5 ± 6.7	(4.6 ± 9.3)	83.6 ± 4.2	81.2 ± 4.8	(2.36 ± 4.6)
Non-randomized controlled trial														
Bunting, Smith, Sutherland [21]	CT (quasi-experimental single arm)	44.4	NA	126.3 ± 14.20		NA	77.8 ± 9.67		NA	137.3 ± 16.85		NA	82.6 ± 11.62	
Erhun, Agbani, Bolaji [22]	CT (quasi-experimental; single arm)	33.3	NA	126.2 ± 6.20		NA	80.6 ± 4.7		NA	167.9 ± 30.32		NA	103.9 ± 30.1	
Aguiar et al. [25]	CT (quasi-experimental; single arm)	40.7	NA	131.8 ± 14.2		NA	77.7 ± 10.4		NA	158.1 ± 15.0		NA	88.1 ± 10.8	
Aguwa, Ukwe, Ekwunife [26]	CT (quasi-experimental; single arm)	44.4	NA	143.8 ± 10.7		NA	89.8 ± 9.7		NA	158.1 ± 14.0		NA	100.6 ± 1.5	
Chabot et al. [31]	CT	66.7	141.0 ± NE	135.2 ± NE	5.8	78.0 ± NE	71.5 ± NE	6.5	139.0 ± NE	139.5 ± NE	−0.5	78.0 ± NE	74.0 ± NE	4
Junior et al. [32]	CT (quasi-experimental; single arm)	40.7	NA	128.0 ± 17.0		NA	74.5 ± 13.0		NA	146.0 ± 19.5		NA	86.5 ± 16.0	
Hussain et al. [36]	CT (quasi-experimental, real-life; treated and not-treated)	66.7	NA	137.0 ± NE		NA	78.0 ± NE		NA	144.0 ± 11.8		NA	82 ± 10.2	
Kicklighter et al. [37]	CT	59.3	166.2 ± 20.0	137.8 ± 23.6	28.4	98.2 ± 11.2	81.7 ± 11.9	16.5	159.9 ± 15.3	143.2 ± 21.0	16.7	90.2 ± 10.8	83.6 ± 10.7	6.6
Parker et al. [40]	CT (quasi-experimental; single arm)	74.1	NA	134.5 ± 14.4		NA	74.0 ± 11.0		NA	146.5 ± 10.9		NA	79.0 ± 10.6	
Taylor et al. [44]	CT (quasi-experimental; single arm)	29.6	NA	141.1 ± 10.5		NA	82 ± 14.4		NA	151.0 ± 13.4		NA	95.6 ± 12.6	
Oparah et al. [53]	CT (quasi-experimental; single arm)	48.1	NA	137.2 ± 21.6		NA	89.0 ± 17.2		NA	187.7 ± 29.5		NA	117.6 ± 21.6	
Robinson et al. [57]	CT	74.1	151.5 ± 14.0	141.6 ± NE	9.9	82.4 ± 13.2	79.5 ± NE	2.9	151.5 ± 14.9	148.7 ± NE	2.8	87.4 ± 9.9	86.4 ± NE	1
Cazarim et al. [60]	CT (quasi-experimental; single arm)	63.0	NA	119.7 ± 7.3		NA	76.7 ± 5.8		NA	137.3 ± 20.1		NA	85.2 ± 11.5	
Mean ± SD			152.9 ± 12.6	133.9 ± 7.1	(14.7 ± 12.0)	86.2 ± 10.6	79.4 ± 5.3	(8.6 ± 7.0)	150.1 ± 10.5	151.2 ± 14.1	(6.3 ± 9.1)	85.2 ± 6.4	89.6 ± 11.8	(3.9 ± 2.8)
Observational studies														
Bex et al. [27]	Observational (descriptive: before/after)	29.6	NA	130.1 ± 13.8		NA	74.5 ± 10.3		NA	152.9 ± 16.5		NA	85.8 ± 11.3	
Zillich et al. [50]	Observational (case–control)	63.0	139.9 ± 11.0	132.8 ± 11.0	7.1	80.0 ± 9.3	76.8 ± 9.3	3.2	136.7 ± 10.6	134.1 ± 10.6	2.6	78.2 ± 8.7	77.0 ± 8.7	1.2
Erickson et al. [54]	Observational (prospective cohort)	63.0	156.5 ± NE	144.5 ± NE	12.0	91.6 ± NE	86.9 ± NE	4.7	153.7 ± NE	151 ± NE	2.7	90.4 ± NE	87.8 ± NE	2.6
Mean ± SD			148.2 ± 11.7	138.6 ± 8.3	(9.5 ± 3.5)	85.8 ± 8.2	81.8 ± 7.1	(3.9 ± 1.0)	145.2 ± 12.0	142.5 ± 11.9	(2.6 ± 0.1)	84.3 ± 8.6	82.4 ± 7.6	(1.9 ± 0.1)

Caption: SBP= systolic blood pressure; DBP = diastolic blood pressure; NA = not applied; NE = not specified; SD = Standard Deviation; * negative difference between baseline and endpoint.

**Table 7:** Application of PI(E)COS acronym for the search strategy.

PI(E)CO Acronym	Description
P—population	Patients undergoing treatment for systemic arterial hypertension.
I—intervention	MTM/pharmaceutical care.
E—exposition	Unexposed individuals are those who are assisted by pharmaceutical care and exposed, those who received only conventional care from the health system.
C—comparison	Conventional care for hypertensive patients in primary care (all services offered by the health system in this area without pharmaceutical care).
O—outcome	Not applicable.
S—study design	Clinical trials and observational studies.

## Data Availability

Data are available upon request and provided by the corresponding author. The protocol of this systematic review was registered in the PROSPERO database and updated on 30 March 2023 [protocol number: is CRD42017079761]. Data can be viewed from the literature, since this is a systematic review; analyzed data can be requested from the corresponding author.

## Supplementary Materials

The following supporting information can be downloaded at: https://www.mdpi.com/article/10.3390/ph16060845/s1, Table S1: scientific studies/documents that were excluded from the systematic review; Table S2: Emtree terms for the search strategy in each database and; Item S1: search strategy combined for each database.
