# Supplementary material for: Effect of Medication Therapy Management by Pharmaceutical Care on Blood Pressure and Cardiovascular Risk in Hypertension: A Systematic Review, Meta-Analysis, and Meta-Regression
Source: Pharmaceuticals (Basel). 2023 Jun 6;16(6):845. doi: 10.3390/ph16060845 (PMC10302324; doi:10.3390/ph16060845)
Supplement: Supplementary file 1 [file pharmaceuticals-16-00845-s001.zip › pharmaceuticals-2346905-supplementary.pdf]

Supplementary Materials

Table S1- scientific studies/documents that were excluded from the systematic review.

Recovered studies (eligible for full text)	Reason of exclusion
AGUIAR, P. M.; BALISA-ROCHA, B. J.; BRITO, G. D.; SILVA, W. B.; MACHADO, M.; LYRA-JR, D. P. Pharmaceutical care in hypertensive patients: a systematic literature review. **Research in Social and Administrative Pharmacy**. v. 8, n. 5, p. 383-96, 2012.	Systematic Review
ALCANTARA, A. P.; TERRA JUNIOR, A. T. Pharmaceutical intervention at the primary Health Care Family. **Revista Cientifica da Faculdade de Educação e Meio Ambiente.** v. 7; n. 2; p. 13-32, 2016.	Integrative Review
AMARILES, P.; SABATER-HERNÁNDEZ, D.; GARCÍA-JIMÉNEZ, E.; RODRÍGUEZ-CHAMORRO, M. Á.; PRATS-MÁS, R.; MARÍN-MAGÁN, F.; et al. Effectiveness of Dader Method for pharmaceutical care on control of blood pressure and total cholesterol in outpatients with cardiovascular disease or cardiovascular risk: EMDADER-CV randomized controlled trial. **Journal of Managed Care Pharmacy**. v. 18; n. 4; p. 311-23, 2012.	Not necessarily treating hypertensive patients
ARIAS, J. L.; SANTAMARIA-LOPEZ, J. M. Improvement of drug therapy results in patients with high blood pressure at a community pharmacy. ARS Pharmaceutica. v. 49; p. 13-24, 2008.	Describes outcomes/program and does not assess outcomes
BRITO, G. C.; MENEZES, M. S.; MESQUITA, A. R.; LYRA-JUNIOR, D. P. Effect of a drug therapy management program in a group of elderly patients with hypertension in Aracaju (Sergipe, Brazil). **Journal of Basic and Applied Pharmaceutical Sciences**. v. 30; n. 1; p. 83-9, 2009.	Describes outcomes/program and does not assess outcomes
CARTER, B. L.; ARDERY, G.; DAWSON, J. D.; JAMES, P. A.; BERGUS, G. R.; DOUCETTE, W. R.; et al. Physician and pharmacist collaboration to improve blood pressure control. **Archives Of Internal Medicine**. v. 169; n. 21; p. 1996-2002, 2009.	There is not necessarily contact with the patient
CARTER, B. L.; ZILLICH, A. J.; ELLIOTT, W. J. How pharmacists can assist physicians with controlling blood pressure. **The Journal of Clinical Hypertension**. v. 5; n. 1; p. 31-7, 2003.	Narrative Review

CASTRO, M. S.; CHEMELLO, C.; PILGER, D.; JUNGES, F.; BOHNEN, L.; ZIMMERMAN, L. M.; et al. Contribuição da atenção farmacêutica no tratamento de pacientes hipertensos. **Revista Brasileira de Hipertensão**. v. 13; n. 3.; p. 198-202, 2006.	Narrative Review
CASTRO, M. S.; et al. Effectiveness of a Pharmaceutical Care Program on Primary Care Patients With Uncontrolled Hypertension- A Brazilian Multicenter RCT. PROTOCOLO DE PESQUISA. 2008. Universidade Federal do Rio Grande do Sul, Porto Alegre-RS, Brazil. Disponível: www.ClinicalTrials.gov. Identifier: NCT00807131	Clinical protocol
CASTRO, M. S.; FUCHS, F. D.; COSTA, S. M.; MAXIMILIANO, P.; GUS, M.; MOREIRA, B. L.; FERREIRA, M. B. F. Pharmaceutical care program for patients with uncontrolled hypertension: report of a double-blind clinical trial with ambulatory blood pressure monitoring. **American journal of hypertension**.v.19, n. 5, p. 528-33, 2006.	Methodology does not qualify for review
CHEEMA, E.; SUTCLIFFE, P.; SINGER, D. R. The impact of interventions by pharmacists in community pharmacies on control of hypertension: a systematic review and meta-analysis of randomized controlled trials. **British journal of clinical pharmacology**. v. 78, n. 6, p. 1238-47, 2014.	Systematic Review
CHEN, X. J.; GAO, X. L.; YOU, G. Y.; JIANG, J.; SUN, X. L.; LI, X.; et al. Higher blood pressure control rate in a real life management program provided by the community health service center in China. **BMC Public Health**. v. 14; n. 1; p. 801, 2014.	Intervention not performed by pharmacists
DONG, B. J.; ECHAVES, S. A.; BRODY, R. V.; SHAPIRO, D. W. Pharmacist provision of preventive health care services in a hypertension clinic. **American Journal of Health System Pharmacy**. v. 54; n. 5; p. 564-6, 1997.	Case report
FIKRI-BENBRAHIM, N.; FAUS, M. J.; MARTÍNEZ-MARTÍNEZ, F.; ALSINA, D. G.; SABATER-HERNÁNDEZ, D. Effect of a pharmacist intervention in Spanish community pharmacies on blood pressure control in hypertensive patients. **American Journal of Health-System Pharmacy.** v. 69, n. 15, p. 1311-8, 2022.	Methodology does not qualify for review
FIKRI-BENBRAHIM, N.; SABATER-HERNÁNDEZ, D.; FIKRI-BENBRAHIM, O.; FAUS-DÁDER, M., J.; MARTÍNEZ-MARTÍNEZ, F.; GONZÁLEZ-SEGURA ALSINA, D. Effect of pharmaceutical intervention on medication adherence and blood pressure	Describes outcomes/program and does not assess outcomes

control in treated hypertensive patients: rationale, design and methods of the AFenPA pilot study. **Ars Pharmaceutica**. v. 52; n. 4; p. 29-38, 2011.	
GUMS, T. H.; URIBE, L.; VANDER, M. W.; JAMES, P.; COFFEY, C.; CARTER, B. L. Pharmacist intervention for blood pressure control: medication intensification and adherence. **Journal of the American Society of Hypertension**. v. 9, n. 7, p.569-78, 2015.	does not assess outcomes for this review
HALE, K.; et al. First-year results of a community pharmacy-based hypertension self-management education program. PÔSTER. **Journal of American Pharmacists Association**. v. 53, Issue 2, p. e8–e117, 2013. DOI: 10.1331/JAPhA.2013.13508	Published abstract
HIRSCH, J. D.; STEERS, N.,;ADLER, D. S.; KUO, G. M.; MORELLO, C. M.; LANG, M.; SINGH, R. F. A randomized pragmatic trial of primary care based pharmacist-physician collaborative medication therapy management for hypertension. **Clinical therapeutics**. v. 36, n. 9, p.1244, 2014.	Consider interventions at the tertiary level
HOULE, S. K.; CHUCK, A. W.; MCALISTER, F. A.; TSUYUKI, R. T. Effect of a Pharmacist-Managed Hypertension Program on Health System Costs: An Evaluation of the Study of Cardiovascular Risk Intervention by Pharmacists—Hypertension (SCRIP-HTN). **Pharmacotherapy: The Journal of Human Pharmacology and Drug Therapy**. v. 32; n. 6; p. 527-37, 2012.	Methodology does not qualify for review
ISETTS, B. J.; BUFFINGTON, D. E.; CARTER, B. L.; SMITH, M.; POLGREEN, L. A.; JAMES, P. A. Evaluation of pharmacists' work in a physician-pharmacist collaborative model for the management of hypertension. **Pharmacotherapy: The Journal of Human Pharmacology and Drug Therapy.** v. 36; n. 4; p. 373-84, 2016.	Methodology does not qualify for review
JACKSON, G. L.; WEINBERGER, M.; KIRSHNER, M. A.; STECHUCHAK, K. M.; MELNYK, S. D.; BOSWORTH, H. B.; et al. Open-label randomized trial of titrated disease management for patients with hypertension: Study design and baseline sample characteristics. **Contemporary Clinical Trials.** v. 50; p. 5-15, 2016.	There is not necessarily contact with the patient
KHEIR, N.; AWAISU, A.; SHARFI, A.; KIDA, M.; ADAM, A. Drug-related problems identified by pharmacists conducting medication use reviews at a primary health center in Qatar. **International journal of clinical pharmacy**. v. 36; n. 4; p. 702-6, 2014.	It does not evaluate the outcomes of the review

LEE, J. K.; GRACE, K. A.; TAYLOR, A. J. Effect of a pharmacy care program on medication adherence and persistence, blood pressure, and low-density lipoprotein cholesterol: a randomized controlled trial. **JAMA.** v. 296; n. 21; p. 2563-71, 2006.	Not necessarily treating hypertensive patients
LUQUE, R.; MARTÍNEZ-MARTÍNEZ, F.; MARTÍ, M.; GASTELURRUTIA, M. A.; DAGO, A.; ANDRÉS, J. Systematic review of the spanish studies about community pharmaceutical care in hypertension. **Pharmaceutical Care Espana**. v. 16, n. 5, p. 193-202, 2013.	Systematic Review
LUQUE, R.; SUAREZ, B.; MARTINEZ-MARTINEZ, F.; GASTELURRUTIA, M. A.; MARTI, M.; ANDRES, J. Effectiveness of pharmaceutical care in medicated hypertensive patients: AFPRES CLM Study. *In* **International Journal of Clinical Pharmacy**. v. 38; n. 4; p. 1018-9, 2016.	Published abstract
MACHADO, M; BAJCAR, J; GUZZO, G. C.; EINARSON, T. R. Sensitivity of patient outcomes to pharmacist interventions. Part II: Systematic review and meta-analysis in hypertension management. **Ann. Pharmacother.,** v. 41, n. 11, p. 1170-1181, 2007.	Systematic Review
MARFO, A. F.; OWUSU-DAAKU, F. T. Evaluation of a pharmacist-led hypertension preventative and detection service in the Ghanaian community pharmacy: an exploratory study. **International Journal of Pharmacy Practice**. v. 24; n. 5; p. 341-8, 2016.	Methodology does not qualify for review
MARTINS, B. P. R.; AQUINO, A. T. D.; PROVIN, M. P.; LIMA, D. M.; DEWULF, N. D. L. S.; AMARAL, R. G. Pharmaceutical care for hypertensive patients provided within the family health strategy in Goiânia, Goiás, Brazil. **Brazilian Journal of Pharmaceutical Sciences**. v. 49; n. 3; p. 609-18, 2013.	It does not evaluate the outcomes of the review
MCKENNEY, J. M.; SLINING, J. M.; HENDERSON, H. R.; DEVINS, D.; BARR, M. The effect of clinical pharmacy services on patients with essential hypertension. **Circulation**. v. 48; n. 5; p. 1104-11, 1973.	Pharmaceutical service does not qualify for review
McNAMARA, K. P.; O'REILLY, S. L.; DUNBAR, J. A.; BAILEY, M. J.; GEORGE, J.; PETERSON, G. M.; et al. A pilot study evaluating multiple risk factor interventions by community pharmacists to prevent cardiovascular disease: the PAART CVD pilot project. **Annals of Pharmacotherapy.** v. 46; n. 2; p. 183-91, 2012.	Not necessarily treating hypertensive patients

MENDONÇA, S. A. M.; MELO, A. C.; PEREIRA, G. C. C.; SANTOS, D. M. S. S.; GROSSI, E. B.; SOUSA, M. C. V. B.; OLIVEIRA, D. R.; SOARES, A. C. Brazilian Journal of Pharmaceutical Sciences. v. 52; n. 3; p. 365-73, 2016.	Assess the service, not patient outcomes
MINO-LÉON, D.; REYES-MORALES, H.; FLORES-HERNÁNDEZ, S. Effectiveness of involving pharmacists in the process of ambulatory health care to improve drug treatment adherence and disease control. **Journal of evaluation in clinical practice**. v. 21; n. 1; p. 7-12, 2015.	Not necessarily treating hypertensive patients
MORGADO, M. P.; MORGADO, S. R.; MENDES, L. C.; PEREIRA, L. J.; CASTELO-BRANCO, M. Pharmacist interventions to enhance blood pressure control and adherence to antihypertensive therapy: review and meta-analysis. **American Journal of Health-System Pharmacy**. v. 68, n. 3, p. 241-53, 2011.	Systematic Review
NETO, P. R. O.; MARUSIC, S.; LYRA-JUNIOR, D. P.; PILGER, D.; CRUCIOL-SOUZA, M. J.; PENTEADO-GAETI, W.; NAKAMURA-CUMAN, R. K. Effect of a 36-month pharmaceutical care program on the coronary heart disease risk in elderly diabetic and hypertensive patients. **Journal of Pharmacy and Pharmaceutical Sciences**. v. 14; n. 2; p. 249-63, 2021.	Not necessarily treating hypertensive patients
NI, Y.; CHEN, Y.; HUANG, W. The effect of pharmaceutical care programs on blood pressure control in individuals with hypertension: A meta-analysis. **Journal of Pharmacy Technology**. v. 25; n. 5; p. 292-6, 2009.	Published abstract
OBRELI-NETO, P. R.; GUIDONI, C. M.; BALDONI, A. O.; PILGER, D.; CRUCIOL-SOUZA, J. M.; GAETI-FRANCO, W. P.; CUMAN, R. K. N. Effect of a 36-month pharmaceutical care program on pharmacotherapy adherence in elderly diabetic and hypertensive patients. **International Journal of Clinical Pharmacy.** v. 33; n. 4; p. 642-9, 2011.	Not necessarily treating hypertensive patients
OBRELI-NETO, P. R.; MARUSIC, S.; GUIDONI, C. M.; BALDONI, A. O.; RENOVATO, R. D.; PILGER, D.; PEREIRA, L. R. L. Economic evaluation of a pharmaceutical care program for elderly diabetic and hypertensive patients in primary health care: a 36-month randomized controlled clinical trial. **Journal of Managed Care & Specialty Pharmacy**. v. 21; n. 1; p. 66-75, 2015.	Not necessarily treating hypertensive patients
PAPADAKIS, E.; et al. The role of pharmacy-based health promotion programs in combating hypertension: the experience of a large community-based pharmacy	Published abstract

in Greece. POSTER AND ORAL PRESENTATION. **World Congress of the International Pharmaceutical Federation**. 2011. Disponível: <https://213.206.87.16/abstracts?page=abstracts&action=item&item=6378 >.	
POLGREEN, L. A.; HAN, J.; CARTER, B. L.; ARDERY, G. P.; COFFEY, C. S.; CHRISCHILLES, E. A.; JAMES, P. A. Cost-effectiveness of a physician–pharmacist collaboration intervention to improve blood pressure control. **Hypertension.** v. 66; n. 6; p. 1145-51, 2015.	It does not evaluate the outcomes of the review
PROVIN, M. P.; CAMPOS, A. D. P.; NIELSON, S. E. D. O.; AMARAL, R. G. Pharmaceutical Care in Goiânia: inclusion of the pharmacist in the Family Health Strategy. **Saúde e Sociedade.** v. 19; n. 3; p. 717-24, 2010.	Methodology does not qualify for review
SANTSCHI, V.; CHIOLERO, A.; BURNAND, B.; COLOSIMO, A. L.; PARADIS, G. Impact of pharmacist care in the management of cardiovascular disease risk factors: a systematic review and meta-analysis of randomized trials. **Archives of internal medicine**. v. 171, n. 16, p. 1441-53, 2011.	Systematic Review
SANTSCHI, V.; CHIOLERO, A.; COLOSIMO, A. L.; PLATT, R. W.; TAFFÉ, P.; BURNIER, M.; et al. Improving blood pressure control through pharmacist interventions: a meta-analysis of randomized controlled trials. **Journal of the American Heart Association**. v. 3; n. 2; p. e000718, 2014.	It does not evaluate the outcomes of the review
SHOJI, M.; OKADA, H.; ONDA, M.; SAKANE, N.; NAKAYAMA, T. PCV142 - Effect of intervention by community pharmacists on awareness of continuing treatment among patients with hypertension. **Value in Health.** v. 18; n. 7; p. A399, 2015.	Published abstract
SUN, J.; et al. The influence of pharmaceutical care dominated by pharmacists on medication of community patients with hypertension. PÔSTER. **Pharmaceutical Care and Research,** v. 12, n. 3, p.180-184, 2012. DOI: 10.5428/pcar20120306	Published abstract
SVARSTAD, B. L.; KOTCHEN, J. M.; SHIREMAN, T. I.; CRAWFORD, S. Y.; PALMER, P. A.; VIVIAN, E. M.; BROWN, R. L. The Team Education and Adherence Monitoring (TEAM) trial: pharmacy interventions to improve hypertension control in blacks. **Circulation: Cardiovascular Quality and Outcomes**. v. 2; n. 3; p. 264-71, 2009.	Clinical protocol

WENTZLAFF, D. M.; CARTER, B. L.; ARDERY, G.; FRANCISCUS, C. L.; DOUCETTE, W. R.; CHRISCHILLES, E. A.; et al. Sustained blood pressure control following discontinuation of a pharmacist intervention. **The Journal of Clinical Hypertension.** v. 13; n. 6; p. 431-7, 2011.	There is not necessarily contact with the patient
ZARAGOZA-FERNÁNDEZ, P.; GASTELURRUTIA, M. Á.; CARDERO, M.; MARTÍNEZ-MARTÍNEZ, F. Intensive two-month intervention on diet and lifestyle in uncontrolled hypertensive patients in a community pharmacy. **Latin American Journal of Pharmacy.** v. 31; n. 5; p. 727-33, 2012.	Published abstract
ZILLICH, A. J.; SUTHERLAND, J. M.; KUMBERA, P. A.; CARTER, B. L. Hypertension outcomes through blood pressure monitoring and evaluation by pharmacists (HOME study). **Journal of General Internal Medicine.** v. 20; n. 12; p. 1091-6, 2005.	Assess the service, not patient outcomes

These 48 excluded studies were classified into the following exclusion categories:classification of exclusion of studies: Reason 1- Non-pharmaceutical interventions (1); Reason 2- Evaluates the service and not outcomes (; Reason 3- Does not assess the outcomes of the review; Reason 4- The service is not suitable for review/intervention at a tertiary level; Reason 5- describes the results of the program; Reason 6- The methodology does not fit the review criteria; Reason 7- There was not necessarily contact with the patient; Reason 8- It is not necessarily a hypertensive patient; Reason 9- Clinical protocol, published abstract, case report and review.

Table S2 – Emtree terms for the search strategy in each database.

MeSH	Emtree thesaurus	DeCS
**Population:** "hypertension"[MeSH Terms] OR "Blood Pressure"[MeSH Terms] OR "Arterial Pressure"[MeSH Terms]	**População:** "hypertension"/exp OR "blood pressure"/exp OR "arterial pressure"/exp OR "pulse pressure"/exp OR "diastolic blood pressure"/exp OR "systolic blood pressure"/exp	**Population:** (Hypertension) OR (Prehypertension) OR (Blood AND Pressure) OR (Arterial AND Pressure) OR (Pulse) OR (Blood AND Pressure AND High) OR (Hipertensión) OR (Prehipertensión) OR (Presión AND Sanguínea) OR (Presión AND Arterial) OR (Pulso AND Arterial) OR (Presión arterial alta) OR (Hipertensão) OR (Pré-Hipertensão) OR (Pressão AND Sanguínea) OR (Pressão AND Arterial) OR (Pressão AND Arterial AND Alta) OR (Diastolic AND Pressure) OR (Presión AND Diastólicas) OR (Pressão AND Diastólica) OR (Pressure AND Systolic) OR (Presión AND Sistólica) OR (Pressão AND Sistólica)
**Intervention:** "Pharmacy"[MeSH Terms] OR "Pharmacists"[MeSH Terms] OR "Pharmaceutical Services"[MeSH Terms]	**Intervenção:** "pharmaceutical care"/exp OR "clinical pharmacy"/exp OR "pharmacist"/exp	**Intervention:** (Pharmaceutical AND Care) OR (Atención AND Farmacéutica) OR (Atenção AND Farmacêutica) OR (Cuidado AND Farmacêutico) OR (Community AND Pharmacy AND Services) OR (Servicios AND Comunitario AND de AND Farmacia) OR (Serviços AND Comunitário AND de AND Farmácia) OR (Pharmaceutical AND Services) OR (Servicios AND Farmacéuticos) OR (Assistência AND Farmacêutica) OR (Pharmacists) OR (Farmacéutico) OR (Farmacêutico) OR (Pharmacy) OR (Farmacia) OR (Farmácia) OR (Pharmacy AND Residencie) OR (Residencia AND en AND Farmacia) OR (Residência AND em AND Farmácia) OR (Medication AND Therapy AND Management) OR (Administración AND de AND Terapia AND de AND Medicación) OR (Conduta AND do AND Tratamento AND Medicamentoso) OR (Drug AND Therapy AND Management) OR (Gestión AND de AND Terapia AND de AND Medicamentos) OR (Gerenciamento AND da AND terapia AND medicamentosa) OR (Medication AND Reconciliation) OR (Conciliación AND de AND Medicamentos) OR (Reconciliação AND de AND Medicamentos) OR (Pharmacist AND Intervention) OR (intervención AND Farmacéutica) OR (Intervenção AND Farmacêutica)
**Comparator:** "Primary Health Care"[MeSH Terms] OR "Community Health Services"[MeSH Terms] OR "Preventive Medicine"[MeSH	**Comparação:** "Primary Health Care"/exp OR "Community Care"/exp OR "Preventive	**Comparator:** (Primary AND Health AND Care) OR (Atención AND Primaria AND de AND Salud) OR (Atenção AND Primária AND à AND Saúde) OR (Primary AND Care) OR (Atención AND Primaria) OR (Atenção AND Primária) OR (Atenção AND Básica) OR (Community AND Health AND Services) OR (Servicios AND de AND Salud AND Comunitaria) OR (Serviços AND de AND Saúde AND Comunitária) OR (Community AND

Terms] OR “Secondary Care"[MeSH Terms]	Medicine"/exp OR “Secondary Health Care"/exp	Health AND Care) OR (Cuidado AND de AND Salud AND Comunitario) OR (Saúde AND Comunitária) OR (Preventive AND Medicine) OR (Medicina AND Preventiva) OR (Comprehensive AND Health AND Care) OR (Atención AND Integral AND de AND Salud) OR (Assistência AND Integral AND à AND Saúde) OR (Secondary AND Care) OR (Atención AND Secundaria AND de AND Salud) OR (Atenção AND Secundária AND à AND Saúde) OR (Secondary AND Health AND Care) OR (Cuidado AND de AND la AND Salud AND secundaria) OR (Cuidados AND de AND saúde AND secundários)

Item S1 – search strategy combined for each database

PubMed, Cochrane, Web of Sciences and Scopus

PUBMED

Population Search: 878511

(("hypertension"[MeSH Terms] OR "Blood Pressure"[MeSH Terms] OR “Arterial Pressure”[MeSH Terms])) OR ((hypertension*) OR (“Blood Pressure High”) OR (“Blood Pressures High”) OR (“High Blood Pressure”) OR (“High Blood Pressures”) OR (“Pressure Blood”) OR (“Blood Pressure”) OR (“Pressures Blood”) OR (“Blood Pressures”) OR (“Diastolic Pressure”) OR (“Pressure Diastolic”) OR (“Diastolic Pressures”) OR (“Pressures Diastolic”) OR (“Pulse Pressure”) OR (“Pressure Pulse”) OR (“Systolic Pressure”) OR (“Pulse Pressures”) OR (“Pressures Pulse”) OR (“Systolic Pressures”) OR (“Pressure Systolic”) OR (“Pressures Systolic”) OR (“Arterial Pressure”) OR (“Arterial Pressures”) OR (“Pressure Arterial”) OR (“Pressures Arterial”) OR (“Arterial Tension”) OR (“Arterial Tensions”) OR (“Tension Arterial”) OR (“Tensions Arterial”) OR (“Blood Pressure Arterial”) OR (“Arterial Blood Pressure”) OR (“Arterial Blood Pressures”) OR (“Blood Pressures Arterial”) OR (“Pressure Arterial Blood”) OR (“Pressures Arterial Blood”) OR (“Aortic Pulse Pressure”) OR (“Aortic Pulse Pressures”) OR (“Pressure Aortic Pulse”) OR (“Pressures Aortic Pulse”) OR (“Pulse Pressure Aortic”) OR (“Pulse Pressures Aortic”) OR (“Mean Arterial Pressure”) OR (“Arterial Pressure Mean”) OR (“Arterial Pressures Mean”) OR (“Mean Arterial Pressures”) OR (“Pressure Mean Arterial”) OR (“Pressures Mean Arterial”) OR (“Aortic Pressure”) OR (“Aortic Pressures”) OR (“Pressure Aortic”) OR (“Pressures Aortic”) OR (“Aortic Tension”) OR (“Aortic Tensions”) OR (“Tension Aortic”) OR (“Tensions Aortic”) OR (“Blood Pressure Aortic”) OR (“Aortic Blood Pressure”) OR (“Aortic Blood Pressures”) OR (“Blood Pressures Aortic”) OR (“Pressure Aortic Blood”) OR (“Pressures Aortic Blood”) OR (“Mean Aortic Pressure”) OR (“Aortic Pressure Mean”) OR (“Aortic Pressures Mean”) OR (“Mean Aortic Pressures”) OR (“Pressure Mean Aortic”) OR (“Pressures Mean Aortic”) OR (hypertensive*) OR (“hypertensive patients”) OR (“hypertensive patient”) OR (“mean artery pressure”) OR (“mean blood pressure”) OR (“mean arterial blood pressure”) OR (“mean artery pressure”) OR (“preexistent hypertension”) OR (“hypertensive effect”) OR (“hypertensive response”) OR (“cardiovascular hypertension”) OR (“blood tension”) OR (“blood tensions”) OR (“vascular pressure”) OR (“vascular pressures”) OR (“arterial pulse pressure”) OR (“arterial pulse pressures”) OR (“artery pulse pressure”) OR (“artery pulse pressures”) OR (“pulse tension”) OR (“pulse tensions”) OR (“artery blood pressure”) OR (“artery blood pressures”) OR (“artery pressure”) OR (“artery pressures”) OR (“aorta blood pressure”) OR (“blood pressure aorta”) OR (“pressure aorta”) OR (“aorta blood pressures”) OR (“blood pressures aorta”) OR (“systemic arterial pressure”) OR (“systemic artery pressure”) OR ("diastolic blood pressure") OR ("Systolic blood pressure") OR (“blood pressure systolic”) OR (“blood systolic pressure”) OR (“blood diastolic pressure”) OR (“blood pressure diastolic”) OR (“systemic arterial pressures”) OR (“systemic artery pressures”) OR ("diastolic blood pressures") OR ("Systolic blood pressures") OR (“blood pressures systolic”) OR (“blood systolic pressures”) OR (“blood diastolic pressures”) OR (“blood pressures diastolic”))

Intervention Search: 382618

((“Pharmacy"[MeSH Terms] OR “Pharmacists”[MeSH Terms] OR “Pharmaceutical Services”[MeSH Terms])) OR ((“Management Medication Therapy”) OR (“Therapy Management Medication”) OR (“Drug Therapy Management”) OR (“Management Drug Therapy”) OR (“Therapy Management Drug”) OR (“Pharmaceutical Care”) OR (“Care Pharmaceutical”) OR (“Pharmacist Interventions”) OR (“Pharmacist Intervention”) OR (“Pharmacists Interventions”) OR (“Pharmacists Intervention”) OR (“medication management”) OR (“medication reconciliation”) OR (“Pharmaceutic Intervention”) OR (“Pharmaceutic Interventions”) OR (“Pharmaceutical Interventions”) OR (“Pharmaceutical Intervention”) OR (“Pharmaceuticals Intervention”) OR (“Pharmaceuticals Interventions”) OR (“Clinical Pharmacists”) OR (“Clinical

Pharmacist”) OR (“Pharmacist Clinical”) OR (“Pharmacists Clinical”) OR (“Pharmaceutical Service Community”) OR (“Pharmaceutical Services Community”) OR (“Service Community Pharmaceutical”) OR (“Services Community Pharmaceutical”) OR (“Pharmacy Services Community”) OR (“Community Pharmacy Service”) OR (“Pharmacy Service Community”) OR (“Services Community Pharmaceutic”) OR (“Services Community Pharmacy”) OR (“Community Pharmaceutic Services”) OR (“Community Pharmaceutic Service”) OR (“Pharmaceutic Service Community”) OR (“Pharmaceutic Services Community”) OR (“Service Community Pharmaceutic”) OR (“Community Pharmaceutical Services”) OR (“Community Pharmaceutical Service”) OR (“Service Community Pharmacy”) OR (“Pharmacy Service Clinical”) OR (“Service Clinical Pharmacy”) OR (“Clinical Pharmacy Services”) OR (“Pharmacy Services Clinical”) OR (“Services Clinical Pharmacy”) OR (“Clinical Pharmacy Service”) OR (“Pharmacy Residencies”) OR (“Pharmacy Residency”) OR (“Medication Management”) OR (“medication reconciliation”))

Comparator Search: 599715
((“Primary Health Care"[MeSH Terms] OR “Community Health Services"[MeSH Terms] OR “Preventive Medicine"[MeSH Terms] OR “Secondary Care"[MeSH Terms])) OR ((“Care Primary Health”) OR (“Primary Care”) OR (“Care Primary”) OR (“Primary Healthcare”) OR (“Healthcare Primary”) OR (“Health Services Community”) OR (“Community Health Service”) OR (“Health Service Community”) OR (“Service Community Health”) OR (“Services Community Health”) OR (“Community Health Care”) OR (“Care Community Health”) OR (“Health Care Community”) OR (“Community Healthcare”) OR (“Community Healthcares”) OR (“Healthcare Community”) OR (“Healthcares Community”) OR (“Community Health Services”) OR (“Preventative Medicine”) OR (“Medicine Preventative”) OR (“Preventive Medicine”) OR (“Preventive Care”) OR (“Care Preventive”) OR (“Preventative Care”) OR (“Care Preventative”) OR (“Health Care Comprehensive”) OR (“Comprehensive Healthcare”) OR (“Healthcare Comprehensive”) OR (“First Line Care”) OR (“Health Care Primary”) OR (“Community Care Service”) OR (“Community Care Services”) OR (“Preventive Health Care”) OR (“Secondary Health Care”) OR (“Secondary Healthcare”) OR (“Care Secondary”) OR (“Secondary Care”) OR (“Secondary Cares”))

Combined

((((("hypertension"[MeSH Terms] OR "Blood Pressure"[MeSH Terms] OR “Arterial Pressure”[MeSH Terms])) OR ((hypertension*) OR (“Blood Pressure High”) OR (“Blood Pressures High”) OR (“High Blood Pressure”) OR (“High Blood Pressures”) OR (“Pressure Blood”) OR (“Blood Pressure”) OR (“Pressures Blood”) OR (“Blood Pressures”) OR (“Diastolic Pressure”) OR (“Pressure Diastolic”) OR (“Diastolic Pressures”) OR (“Pressures Diastolic”) OR (“Pulse Pressure”) OR (“Pressure Pulse”) OR (“Systolic Pressure”) OR (“Pulse Pressures”) OR (“Pressures Pulse”) OR (“Systolic Pressures”) OR (“Pressure Systolic”) OR (“Pressures Systolic”) OR (“Arterial Pressure”) OR (“Arterial Pressures”) OR (“Pressure Arterial”) OR (“Pressures Arterial”) OR (“Arterial Tension”) OR (“Arterial Tensions”) OR (“Tension Arterial”) OR (“Tensions Arterial”) OR (“Blood Pressure Arterial”) OR (“Arterial Blood Pressure”) OR (“Arterial Blood Pressures”) OR (“Blood Pressures Arterial”) OR (“Pressure Arterial Blood”) OR (“Pressures Arterial Blood”) OR (“Aortic Pulse Pressure”) OR (“Aortic Pulse Pressures”) OR (“Pressure Aortic Pulse”) OR (“Pressures Aortic Pulse”) OR (“Pulse Pressure Aortic”) OR (“Pulse Pressures Aortic”) OR (“Mean Arterial Pressure”) OR (“Arterial Pressure Mean”) OR (“Arterial Pressures Mean”) OR (“Mean Arterial Pressures”) OR (“Pressure Mean Arterial”) OR (“Pressures Mean Arterial”) OR (“Aortic Pressure”) OR (“Aortic Pressures”) OR (“Pressure Aortic”) OR (“Pressures Aortic”) OR (“Aortic Tension”) OR (“Aortic Tensions”) OR (“Tension Aortic”) OR (“Tensions Aortic”) OR (“Blood Pressure Aortic”) OR (“Aortic Blood Pressure”) OR (“Aortic Blood Pressures”) OR (“Blood Pressures Aortic”) OR (“Pressure Aortic Blood”) OR (“Pressures Aortic Blood”) OR (“Mean Aortic Pressure”) OR (“Aortic Pressure Mean”) OR (“Aortic Pressures Mean”) OR (“Mean Aortic Pressures”) OR (“Pressure Mean Aortic”) OR (“Pressures Mean Aortic”) OR (hypertensive*) OR (“hypertensive patients”) OR (“hypertensive patient”) OR (“mean artery pressure”) OR (“mean blood pressure”) OR (“mean

arterial blood pressure”) OR (“mean artery pressure”) OR (“preexistent hypertension”) OR (“hypertensive effect”) OR (“hypertensive response”) OR (“cardiovascular hypertension”) OR (“blood tension”) OR (“blood tensions”) OR (“vascular pressure”) OR (“vascular pressures”) OR (“arterial pulse pressure”) OR (“arterial pulse pressures”) OR (“artery pulse pressure”) OR (“artery pulse pressures”) OR (“pulse tension”) OR (“pulse tensions”) OR (“artery blood pressure”) OR (“artery blood pressures”) OR (“artery pressure”) OR (“artery pressures”) OR (“aorta blood pressure”) OR (“blood pressure aorta”) OR (“pressure aorta”) OR (“aorta blood pressures”) OR (“blood pressures aorta”) OR (“systemic arterial pressure”) OR (“systemic artery pressure”) OR ("diastolic blood pressure") OR ("Systolic blood pressure") OR (“blood pressure systolic”) OR (“blood systolic pressure”) OR (“blood diastolic pressure”) OR (“blood pressure diastolic”) OR (“systemic arterial pressures”) OR (“systemic artery pressures”) OR ("diastolic blood pressures") OR ("Systolic blood pressures") OR (“blood pressures systolic”) OR (“blood systolic pressures”) OR (“blood diastolic pressures”) OR (“blood pressures diastolic”)))) AND (((“Pharmacy"[MeSH Terms] OR “Pharmacists”[MeSH Terms] OR “Pharmaceutical Services”[MeSH Terms])) OR ((“Management Medication Therapy”) OR (“Therapy Management Medication”) OR (“Drug Therapy Management”) OR (“Management Drug Therapy”) OR (“Therapy Management Drug”) OR (“Pharmaceutical Care”) OR (“Care Pharmaceutical”) OR (“Pharmacist Interventions”) OR (“Pharmacist Intervention”) OR (“Pharmacists Interventions”) OR (“Pharmacists Intervention”) OR (“medication management”) OR (“medication reconciliation”) OR (“Pharmaceutic Intervention”) OR (“Pharmaceutic Interventions”) OR (“Pharmaceutical Interventions”) OR (“Pharmaceutical Intervention”) OR (“Pharmaceuticals Intervention”) OR (“Pharmaceuticals Interventions”) OR (“Clinical Pharmacists”) OR (“Clinical Pharmacist”) OR (“Pharmacist Clinical”) OR (“Pharmacists Clinical”) OR (“Pharmaceutical Service Community”) OR (“Pharmaceutical Services Community”) OR (“Service Community Pharmaceutical”) OR (“Services Community Pharmaceutical”) OR (“Pharmacy Services Community”) OR (“Community Pharmacy Service”) OR (“Pharmacy Service Community”) OR (“Services Community Pharmaceutic”) OR (“Services Community Pharmacy”) OR (“Community Pharmaceutic Services”) OR (“Community Pharmaceutic Service”) OR (“Pharmaceutic Service Community”) OR (“Pharmaceutic Services Community”) OR (“Service Community Pharmaceutic”) OR (“Community Pharmaceutical Services”) OR (“Community Pharmaceutical Service”) OR (“Service Community Pharmacy”) OR (“Pharmacy Service Clinical”) OR (“Service Clinical Pharmacy”) OR (“Clinical Pharmacy Services”) OR (“Pharmacy Services Clinical”) OR (“Services Clinical Pharmacy”) OR (“Clinical Pharmacy Service”) OR (“Pharmacy Residencies”) OR (“Pharmacy Residency”) OR (“Medication Management”) OR (“medication reconciliation”)))) AND (((“Primary Health Care"[MeSH Terms] OR “Community Health Services"[MeSH Terms] OR “Preventive Medicine"[MeSH Terms] OR “Secondary Care"[MeSH Terms])) OR ((“Care Primary Health”) OR (“Primary Care”) OR (“Care Primary”) OR (“Primary Healthcare”) OR (“Healthcare Primary”) OR (“Health Services Community”) OR (“Community Health Service”) OR (“Health Service Community”) OR (“Service Community Health”) OR (“Services Community Health”) OR (“Community Health Care”) OR (“Care Community Health”) OR (“Health Care Community”) OR (“Community Healthcare”) OR (“Community Healthcares”) OR (“Healthcare Community”) OR (“Healthcares Community”) OR (“Community Health Services”) OR (“Preventative Medicine”) OR (“Medicine Preventative”) OR (“Preventive Medicine”) OR (“Preventive Care”) OR (“Care Preventive”) OR (“Preventative Care”) OR (“Care Preventative”) OR (“Health Care Comprehensive”) OR (“Comprehensive Healthcare”) OR (“Healthcare Comprehensive”) OR (“First Line Care”) OR (“Health Care Primary”) OR (“Community Care Service”) OR (“Community Care Services”) OR (“Preventive Health Care”) OR (“Secondary Health Care”) OR (“Secondary Healthcare”) OR (“Care Secondary”) OR (“Secondary Care”) OR (“Secondary Cares”)))

#Combined Search for PUBMED: 2608

Cochrane: 272; Web of Sciences: 435; Scopus: 1298

EMBASE

População Search: 1247393

'hypertension'/exp OR 'blood pressure'/exp OR 'arterial pressure'/exp OR 'pulse pressure'/exp OR 'diastolic blood pressure'/exp OR 'systolic blood pressure'/exp OR hypertension*:ab,ti OR (blood:ab,ti AND pressure:ab,ti AND high:ab,ti) OR (blood:ab,ti AND pressures:ab,ti AND high:ab,ti) OR (high:ab,ti AND blood:ab,ti AND pressure:ab,ti) OR (high:ab,ti AND blood:ab,ti AND pressures:ab,ti) OR (pressure:ab,ti AND blood:ab,ti) OR (blood:ab,ti AND pressure:ab,ti) OR (pressures:ab,ti AND blood:ab,ti) OR (blood:ab,ti AND pressures:ab,ti) OR (diastolic:ab,ti AND pressure:ab,ti) OR (pressure:ab,ti AND diastolic:ab,ti) OR (diastolic:ab,ti AND pressures:ab,ti) OR (pressures:ab,ti AND diastolic:ab,ti) OR (pulse:ab,ti AND pressure:ab,ti) OR (pressure:ab,ti AND pulse:ab,ti) OR (systolic:ab,ti AND pressure:ab,ti) OR (pulse:ab,ti AND pressures:ab,ti) OR (pressures:ab,ti AND pulse:ab,ti) OR (systolic:ab,ti AND pressures:ab,ti) OR (pressure:ab,ti AND systolic:ab,ti) OR (pressures:ab,ti AND systolic:ab,ti) OR (arterial:ab,ti AND pressure:ab,ti) OR (arterial:ab,ti AND pressures:ab,ti) OR (pressure:ab,ti AND arterial:ab,ti) OR (pressures:ab,ti AND arterial:ab,ti) OR (arterial:ab,ti AND tension:ab,ti) OR (arterial:ab,ti AND tensions:ab,ti) OR (tension:ab,ti AND arterial:ab,ti) OR (tensions:ab,ti AND arterial:ab,ti) OR (blood:ab,ti AND pressure:ab,ti AND arterial:ab,ti) OR (arterial:ab,ti AND blood:ab,ti AND pressure:ab,ti) OR (arterial:ab,ti AND blood:ab,ti AND pressures:ab,ti) OR (blood:ab,ti AND pressures:ab,ti AND arterial:ab,ti) OR (pressure:ab,ti AND arterial:ab,ti AND blood:ab,ti) OR (pressures:ab,ti AND arterial:ab,ti AND blood:ab,ti) OR (aortic:ab,ti AND pulse:ab,ti AND pressure:ab,ti) OR (aortic:ab,ti AND pulse:ab,ti AND pressures:ab,ti) OR (pressure:ab,ti AND aortic:ab,ti AND pulse:ab,ti) OR (pressures:ab,ti AND aortic:ab,ti AND pulse:ab,ti) OR (pulse:ab,ti AND pressure:ab,ti AND aortic:ab,ti) OR (pulse:ab,ti AND pressures:ab,ti AND aortic:ab,ti) OR (mean:ab,ti AND arterial:ab,ti AND pressure:ab,ti) OR (arterial:ab,ti AND pressure:ab,ti AND mean:ab,ti) OR (arterial:ab,ti AND pressures:ab,ti AND mean:ab,ti) OR (mean:ab,ti AND arterial:ab,ti AND pressures:ab,ti) OR (pressure:ab,ti AND mean:ab,ti AND arterial:ab,ti) OR (pressures:ab,ti AND mean:ab,ti AND arterial:ab,ti) OR (aortic:ab,ti AND pressure:ab,ti) OR (aortic:ab,ti AND pressures:ab,ti) OR (pressure:ab,ti AND aortic:ab,ti) OR (pressures:ab,ti AND aortic:ab,ti) OR (aortic:ab,ti AND tension:ab,ti) OR (aortic:ab,ti AND tensions:ab,ti) OR (tension:ab,ti AND aortic:ab,ti) OR (tensions:ab,ti AND aortic:ab,ti) OR (blood:ab,ti AND pressure:ab,ti AND aortic:ab,ti) OR (aortic:ab,ti AND blood:ab,ti AND pressure:ab,ti) OR (aortic:ab,ti AND blood:ab,ti AND pressures:ab,ti) OR (blood:ab,ti AND pressures:ab,ti AND aortic:ab,ti) OR (pressure:ab,ti AND aortic:ab,ti AND blood:ab,ti) OR (pressures:ab,ti AND aortic:ab,ti AND blood:ab,ti) OR (mean:ab,ti AND aortic:ab,ti AND pressure:ab,ti) OR (aortic:ab,ti AND pressure:ab,ti AND mean:ab,ti) OR (aortic:ab,ti AND pressures:ab,ti AND mean:ab,ti) OR (mean:ab,ti AND aortic:ab,ti AND pressures:ab,ti) OR (pressure:ab,ti AND mean:ab,ti AND aortic:ab,ti) OR (pressures:ab,ti AND mean:ab,ti AND aortic:ab,ti) OR hypertensive*:ab,ti OR (hypertensive:ab,ti AND patients:ab,ti) OR (hypertensive:ab,ti AND patient:ab,ti) OR (mean:ab,ti AND blood:ab,ti AND pressure:ab,ti) OR (mean:ab,ti AND arterial:ab,ti AND blood:ab,ti AND pressure:ab,ti) OR (mean:ab,ti AND artery:ab,ti AND pressure:ab,ti) OR (preexistent:ab,ti AND hypertension:ab,ti) OR (hypertensive:ab,ti AND effect:ab,ti) OR (hypertensive:ab,ti AND response:ab,ti) OR (cardiovascular:ab,ti AND hypertension:ab,ti) OR (blood:ab,ti AND tension:ab,ti) OR (blood:ab,ti AND tensions:ab,ti) OR (vascular:ab,ti AND pressure:ab,ti) OR (vascular:ab,ti AND pressures:ab,ti) OR (arterial:ab,ti AND pulse:ab,ti AND pressure:ab,ti) OR (arterial:ab,ti AND pulse:ab,ti AND pressures:ab,ti) OR (artery:ab,ti AND pulse:ab,ti AND pressure:ab,ti) OR (artery:ab,ti AND pulse:ab,ti AND pressures:ab,ti) OR (pulse:ab,ti AND tension:ab,ti) OR (pulse:ab,ti AND tensions:ab,ti) OR (artery:ab,ti AND blood:ab,ti AND pressure:ab,ti) OR (artery:ab,ti AND blood:ab,ti AND pressures:ab,ti) OR (artery:ab,ti AND pressure:ab,ti) OR (artery:ab,ti AND pressures:ab,ti) OR (aorta:ab,ti AND blood:ab,ti AND pressure:ab,ti) OR (blood:ab,ti AND pressure:ab,ti AND aorta:ab,ti) OR (pressure:ab,ti AND aorta:ab,ti) OR (aorta:ab,ti AND blood:ab,ti AND pressures:ab,ti) OR (blood:ab,ti AND

pressures:ab,ti AND aorta:ab,ti) OR (systemic:ab,ti AND arterial:ab,ti AND pressure:ab,ti) OR (systemic:ab,ti AND artery:ab,ti AND pressure:ab,ti) OR (diastolic:ab,ti AND blood:ab,ti AND pressure:ab,ti) OR (systolic:ab,ti AND blood:ab,ti AND pressure:ab,ti) OR (blood:ab,ti AND pressure:ab,ti AND systolic:ab,ti) OR (blood:ab,ti AND systolic:ab,ti AND pressure:ab,ti) OR (blood:ab,ti AND diastolic:ab,ti AND pressure:ab,ti) OR (blood:ab,ti AND pressure:ab,ti AND diastolic:ab,ti) OR (systemic:ab,ti AND arterial:ab,ti AND pressures:ab,ti) OR (systemic:ab,ti AND artery:ab,ti AND pressures:ab,ti) OR (diastolic:ab,ti AND blood:ab,ti AND pressures:ab,ti) OR (systolic:ab,ti AND blood:ab,ti AND pressures:ab,ti) OR (blood:ab,ti AND pressures:ab,ti AND systolic:ab,ti) OR (blood:ab,ti AND systolic:ab,ti AND pressures:ab,ti) OR (blood:ab,ti AND diastolic:ab,ti AND pressures:ab,ti) OR (blood:ab,ti AND pressures:ab,ti AND diastolic:ab,ti)

Intervention Search: 166347

'pharmaceutical care'/exp OR 'clinical pharmacy'/exp OR 'pharmacist'/exp OR (management:ab,ti AND medication:ab,ti AND therapy:ab,ti) OR (therapy:ab,ti AND management:ab,ti AND medication:ab,ti) OR (drug:ab,ti AND therapy:ab,ti AND management:ab,ti) OR (management:ab,ti AND drug:ab,ti AND therapy:ab,ti) OR (therapy:ab,ti AND management:ab,ti AND drug:ab,ti) OR (medication:ab,ti AND management:ab,ti) OR (medication:ab,ti AND reconciliation:ab,ti) OR (services:ab,ti AND pharmaceutic:ab,ti) OR (services:ab,ti AND pharmacy:ab,ti) OR (pharmaceutic:ab,ti AND services:ab,ti) OR (pharmaceutic:ab,ti AND service:ab,ti) OR (service:ab,ti AND pharmaceutic:ab,ti) OR (services:ab,ti AND pharmaceutical:ab,ti) OR (service:ab,ti AND pharmaceutical:ab,ti) OR (pharmacy:ab,ti AND services:ab,ti) OR (pharmacy:ab,ti AND service:ab,ti) OR (service:ab,ti AND pharmacy:ab,ti) OR (pharmaceutical:ab,ti AND care:ab,ti) OR (care:ab,ti AND pharmaceutical:ab,ti) OR (pharmaceutical:ab,ti AND service:ab,ti AND community:ab,ti) OR (pharmaceutical:ab,ti AND services:ab,ti AND community:ab,ti) OR (service:ab,ti AND community:ab,ti AND pharmaceutical:ab,ti) OR (services:ab,ti AND community:ab,ti AND pharmaceutical:ab,ti) OR (pharmacy:ab,ti AND services:ab,ti AND community:ab,ti) OR (community:ab,ti AND pharmacy:ab,ti AND service:ab,ti) OR (pharmacy:ab,ti AND service:ab,ti AND community:ab,ti) OR (services:ab,ti AND community:ab,ti AND pharmaceutic:ab,ti) OR (services:ab,ti AND community:ab,ti AND pharmacy:ab,ti) OR (community:ab,ti AND pharmaceutic:ab,ti AND services:ab,ti) OR (community:ab,ti AND pharmaceutic:ab,ti AND service:ab,ti) OR (pharmaceutic:ab,ti AND service:ab,ti AND community:ab,ti) OR (pharmaceutic:ab,ti AND services:ab,ti AND community:ab,ti) OR (service:ab,ti AND community:ab,ti AND pharmaceutic:ab,ti) OR (community:ab,ti AND pharmaceutical:ab,ti AND services:ab,ti) OR (community:ab,ti AND pharmaceutical:ab,ti AND service:ab,ti) OR (service:ab,ti AND community:ab,ti AND pharmacy:ab,ti) OR (community:ab,ti AND pharmacy:ab,ti) OR (community:ab,ti AND pharmacist:ab,ti) OR (pharmaceutic:ab,ti AND intervention:ab,ti) OR (pharmaceutic:ab,ti AND interventions:ab,ti) OR (pharmaceutical:ab,ti AND interventions:ab,ti) OR (pharmaceutical:ab,ti AND intervention:ab,ti) OR (pharmaceuticals:ab,ti AND intervention:ab,ti) OR (pharmaceuticals:ab,ti AND interventions:ab,ti) OR (pharmacist:ab,ti AND interventions:ab,ti) OR (pharmacist:ab,ti AND intervention:ab,ti) OR (pharmacists:ab,ti AND interventions:ab,ti) OR (pharmacists:ab,ti AND intervention:ab,ti) OR (pharmacy:ab,ti AND service:ab,ti AND clinical:ab,ti) OR (service:ab,ti AND clinical:ab,ti AND pharmacy:ab,ti) OR (clinical:ab,ti AND pharmacy:ab,ti AND services:ab,ti) OR (pharmacy:ab,ti AND services:ab,ti AND clinical:ab,ti) OR (services:ab,ti AND clinical:ab,ti AND pharmacy:ab,ti) OR (clinical:ab,ti AND pharmacy:ab,ti AND service:ab,ti) OR (clinical:ab,ti AND pharmacy:ab,ti) OR (pharmacy:ab,ti AND clinical:ab,ti) OR (pharmacy:ab,ti AND residencies:ab,ti) OR (pharmacy:ab,ti AND residency:ab,ti)

Comparator Search: 1055100

'primary health care'/exp OR 'community care'/exp OR 'preventive medicine'/exp OR 'secondary health care'/exp OR (care AND primary AND health) OR (primary AND care) OR (care AND

primary) OR (primary AND healthcare) OR (healthcare AND primary) OR (health AND services AND community) OR (community AND health AND service) OR (health AND service AND community) OR (service AND community AND health) OR (services AND community AND health) OR (community AND health AND care) OR (care AND community AND health) OR (health AND care AND community) OR (community AND healthcare) OR (community AND healthcares) OR (healthcare AND community) OR (healthcares AND community) OR (community AND health AND services) OR (preventative AND medicine) OR (medicine AND preventative) OR (preventive AND medicine) OR (preventive AND care) OR (care AND preventive) OR (preventative AND care) OR (care AND preventative) OR (health AND care AND comprehensive) OR (comprehensive AND healthcare) OR (healthcare AND comprehensive) OR (first AND line AND care) OR (health AND care AND primary) OR (community AND care AND service) OR (community AND care AND services) OR (preventive AND health AND care) OR (secondary AND health AND care) OR (secondary AND healthcare) OR (care AND secondary) OR (secondary AND care) OR (secondary AND cares)

Combined search: 1653

LILACs

Population Search: 27011

(Hypertension) OR (Prehypertension) OR (Blood AND Pressure) OR (Arterial AND Pressure) OR (Pulse) OR (Blood AND Pressure AND High) OR (Hipertensión) OR (Prehipertensión) OR (Presión AND Sanguínea) OR (Presión AND Arterial) OR (Pulso AND Arterial) OR (Presión arterial alta) OR (Hipertensão) OR (Pré-Hipertensão) OR (Pressão AND Sanguínea) OR (Pressão AND Arterial) OR (Pressão AND Arterial AND Alta) OR (Diastolic AND Pressure) OR (Presión AND Diastólicas) OR (Pressão AND Diastólica) OR (Pressure AND Systolic) OR (Presión AND Sistólica) OR (Pressão AND Sistólica) OR (Hypertensive$ AND Patient$) OR (Paciente$ AND Hipertenso$) OR (Hipertenso$) [Palavras]

Intervention Search: 15574

(Pharmaceutical AND Care) OR (Atención AND Farmacéutica) OR (Atenção AND Farmacêutica) OR (Cuidado AND Farmacêutico) OR (Community AND Pharmacy AND Service$) OR (Servicio$ AND Comunitario$ AND de AND Farmacia) OR (Serviço$ AND Comunitário$ AND de AND Farmácia) OR (Pharmaceutical AND Service$) OR (Servicio$ AND Farmacéutico$) OR (Assistência AND Farmacêutica) OR (Pharmacist$) OR (Farmacéutico$) OR (Farmacêutico$) OR (Pharmacy) OR (Farmacia) OR (Farmácia) OR (Pharmacy AND Residencie$) OR (Residencia$ AND en AND Farmacia) OR (Residência$ AND em AND Farmácia) OR (Medication AND Therapy AND Management) OR (Administración AND de AND Terapia AND de AND Medicación) OR (Conduta AND do AND Tratamento AND Medicamentoso) OR (Drug AND Therapy AND Management) OR (Gestión AND de AND Terapia AND de AND Medicamentos) OR (Gerenciamento AND da AND terapia AND medicamentosa) OR (Medication AND Reconciliation) OR (Conciliación AND de AND Medicamentos) OR (Reconciliação AND de AND Medicamentos) OR (Pharmacist AND Intervention) OR (intervención AND Farmacéutica) OR (Intervenção AND Farmacêutica) [Palavras]

Comparator Search: 38737

(Primary AND Health AND Care) OR (Atención AND Primaria AND de AND Salud) OR (Atenção AND Primária AND à AND Saúde) OR (Primary AND Care) OR (Atención AND Primaria) OR (Atenção AND Primária) OR (Atenção AND Básica) OR (Community AND Health AND Service$) OR (Servicio$ AND de AND Salud AND Comunitaria) OR (Serviço$ AND de AND Saúde AND Comunitária) OR (Community AND Health AND Care) OR (Cuidado AND de AND Salud AND Comunitario) OR (Saúde AND Comunitária) OR (Preventive AND Medicine) OR (Medicina AND Preventiva) OR (Comprehensive AND Health AND Care) OR (Atención AND Integral AND de AND Salud) OR (Assistência AND Integral AND à AND Saúde) OR (Secondary AND Care) OR (Atención AND Secundaria AND de AND Salud) OR (Atenção AND Secundária AND à AND Saúde) OR (Secondary AND Health AND Care) OR (Cuidado AND de AND la AND Salud AND secundaria) OR (Cuidado$ AND de AND saúde AND secundário$) [Palavras]

(Hypertension) OR (Prehypertension) OR (Blood AND Pressure) OR (Arterial AND Pressure) OR (Pulse) OR (Blood AND Pressure AND High) OR (Hipertensión) OR (Prehipertensión) OR (Presión AND Sanguínea) OR (Presión AND Arterial) OR (Pulso AND Arterial) OR (Presión arterial alta) OR (Hipertensão) OR (Pré-Hipertensão) OR (Pressão AND Sanguínea) OR (Pressão AND Arterial) OR (Pressão AND Arterial AND Alta) OR (Diastolic AND Pressure) OR (Presión AND Diastólicas) OR (Pressão AND Diastólica) OR (Pressure AND Systolic) OR (Presión AND Sistólica) OR (Pressão AND Sistólica) OR (Hypertensive$ AND Patient$) OR (Paciente$ AND Hipertenso$) OR (Hipertenso$) [Palavras] and (Pharmaceutical AND Care) OR (Atención AND Farmacéutica) OR (Atenção AND

Farmacêutica) OR (Cuidado AND Farmacêutico) OR (Community AND Pharmacy AND Service$) OR (Servicio$ AND Comunitario$ AND de AND Farmacia) OR (Serviço$ AND Comunitário$ AND de AND Farmácia) OR (Pharmaceutical AND Service$) OR (Servicio$ AND Farmacéutico$) OR (Assistência AND Farmacêutica) OR (Pharmacist$) OR (Farmacéutico$) OR (Farmacêutico$) OR (Pharmacy) OR (Farmacia) OR (Farmácia) OR (Pharmacy AND Residencie$) OR (Residencia$ AND en AND Farmacia) OR (Residência$ AND em AND Farmácia) OR (Medication AND Therapy AND Management) OR (Administración AND de AND Terapia AND de AND Medicación) OR (Conduta AND do AND Tratamento AND Medicamentoso) OR (Drug AND Therapy AND Management) OR (Gestión AND de AND Terapia AND de AND Medicamentos) OR (Gerenciamento AND da AND terapia AND medicamentosa) OR (Medication AND Reconciliation) OR (Conciliación AND de AND Medicamentos) OR (Reconciliação AND de AND Medicamentos) OR (Pharmacist AND Intervention) OR (intervención AND Farmacéutica) OR (Intervenção AND Farmacêutica) [Palavras] and (Primary AND Health AND Care) OR (Atención AND Primaria AND de AND Salud) OR (Atenção AND Primária AND à AND Saúde) OR (Primary AND Care) OR (Atención AND Primaria) OR (Atenção AND Primária) OR (Atenção AND Básica) OR (Community AND Health AND Service$) OR (Servicio$ AND de AND Salud AND Comunitaria) OR (Serviço$ AND de AND Saúde AND Comunitária) OR (Community AND Health AND Care) OR (Cuidado AND de AND Salud AND Comunitario) OR (Saúde AND Comunitária) OR (Preventive AND Medicine) OR (Medicina AND Preventiva) OR (Comprehensive AND Health AND Care) OR (Atención AND Integral AND de AND Salud) OR (Assistência AND Integral AND à AND Saúde) OR (Secondary AND Care) OR (Atención AND Secundaria AND de AND Salud) OR (Atenção AND Secundária AND à AND Saúde) OR (Secondary AND Health AND Care) OR (Cuidado AND de AND la AND Salud AND secundaria) OR (Cuidado$ AND de AND saúde AND secundário$) [Palavras]

Combined search: 97

<u>Combined search to improve the sensitivity</u>

(Hypertension) OR (Prehypertension) OR (Blood AND Pressure) OR (Arterial AND Pressure) OR (Pulse) OR (Blood AND Pressure AND High) OR (Hipertensión) OR (Prehipertensión) OR (Presión AND Sanguínea) OR (Presión AND Arterial) OR (Pulso AND Arterial) OR (Presión arterial alta) OR (Hipertensão) OR (Pré-Hipertensão) OR (Pressão AND Sanguínea) OR (Pressão AND Arterial) OR (Pressão AND Arterial AND Alta) OR (Diastolic AND Pressure) OR (Presión AND Diastólicas) OR (Pressão AND Diastólica) OR (Pressure AND Systolic) OR (Presión AND Sistólica) OR (Pressão AND Sistólica) OR (Hypertensive$ AND Patient$) OR (Paciente$ AND Hipertenso$) OR (Hipertenso$) [Palavras] and (Pharmaceutical AND Care) OR (Atención AND Farmacéutica) OR (Atenção AND Farmacêutica) OR (Cuidado AND Farmacêutico) OR (Community AND Pharmacy AND Service$) OR (Servicio$ AND Comunitario$ AND de AND Farmacia) OR (Serviço$ AND Comunitário$ AND de AND Farmácia) OR (Pharmaceutical AND Service$) OR (Servicio$ AND Farmacéutico$) OR (Assistência AND Farmacêutica) OR (Pharmacist$) OR (Farmacéutico$) OR (Farmacêutico$) OR (Pharmacy) OR (Farmacia) OR (Farmácia) OR (Pharmacy AND Residencie$) OR (Residencia$ AND en AND Farmacia) OR (Residência$ AND em AND Farmácia) OR (Medication AND Therapy AND Management) OR (Administración AND de AND Terapia AND de AND Medicación) OR (Conduta AND do AND Tratamento AND Medicamentoso) OR (Drug AND Therapy AND Management) OR (Gestión AND de AND Terapia AND de AND Medicamentos) OR (Gerenciamento AND da AND terapia AND medicamentosa) OR (Medication AND Reconciliation) OR (Conciliación AND de AND Medicamentos) OR (Reconciliação AND de AND Medicamentos) OR (Pharmacist AND Intervention) OR (intervención AND Farmacéutica) OR (Intervenção AND Farmacêutica) [Palavras]

Combined search only with Population and Intervention: 909

APA

#1 Population

hypertension OR Blood Pressure OR Arterial Pressure OR Prehypertension OR Arterial Pressure OR Pulse OR Blood Pressure High OR pulse pressure OR diastolic blood pressure OR systolic blood pressure

OR hypertension OR Blood Pressure High OR Blood Pressures High OR High Blood Pressure OR High Blood Pressures OR Pressure Blood OR Blood Pressure OR Pressures Blood OR Blood Pressures OR Diastolic Pressure OR Pressure Diastolic OR Diastolic Pressures OR Pressures Diastolic OR Pulse Pressure OR Pressure Pulse OR Systolic Pressure OR Pulse Pressures OR Pressures Pulse OR Systolic Pressures OR Pressure Systolic OR Pressures Systolic OR Arterial Pressure OR Arterial Pressures OR Pressure Arterial OR Pressures Arterial OR Arterial Tension OR Arterial Tensions OR Tension Arterial OR Tensions Arterial OR Blood Pressure Arterial OR Arterial Blood Pressure OR Arterial Blood Pressures OR Blood Pressures Arterial OR Pressure Arterial Blood OR Pressures Arterial Blood OR Aortic Pulse Pressure OR Aortic Pulse Pressures OR Pressure Aortic Pulse OR Pressures Aortic Pulse OR Pulse Pressure Aortic OR Pulse Pressures Aortic OR Mean Arterial Pressure OR Arterial Pressure Mean OR Arterial Pressures Mean OR Mean Arterial Pressures OR Pressure Mean Arterial OR Pressures Mean Arterial OR Aortic Pressure OR Aortic Pressures OR Pressure Aortic OR Pressures Aortic OR Aortic Tension OR Aortic Tensions OR Tension Aortic OR Tensions Aortic OR Blood Pressure Aortic OR Aortic Blood Pressure OR Aortic Blood Pressures OR Blood Pressures Aortic OR Pressure Aortic Blood OR Pressures Aortic Blood OR Mean Aortic Pressure OR Aortic Pressure Mean OR Aortic Pressures Mean OR Mean Aortic Pressures OR Pressure Mean Aortic OR Pressures Mean Aortic OR (hypertensive*) OR hypertensive patients OR hypertensive patient OR mean artery pressure OR mean blood pressure OR mean arterial blood pressure OR mean artery pressure OR preexistent hypertension OR hypertensive effect OR hypertensive response OR cardiovascular hypertension OR blood tension OR blood tensions OR vascular pressure OR vascular pressures OR arterial pulse pressure OR arterial pulse pressures OR artery pulse pressure OR artery pulse pressures OR pulse tension OR pulse tensions OR artery blood pressure OR artery blood pressures OR artery pressure OR artery pressures OR aorta blood pressure OR blood pressure aorta OR pressure aorta OR aorta blood pressures OR blood pressures aorta OR systemic arterial pressure OR systemic artery pressure OR diastolic blood pressure OR Systolic blood pressure OR blood pressure systolic OR blood systolic pressure OR blood diastolic pressure OR blood pressure diastolic OR systemic arterial pressures OR systemic artery pressures OR diastolic blood pressures OR Systolic blood pressures OR blood pressures systolic OR blood systolic pressures OR blood diastolic pressures OR blood pressures diastolic

#2 Intervention

Pharmacy OR Pharmacists OR Pharmaceutical Services OR Pharmaceutical Care OR Community Pharmacy Services OR Pharmacy Residencies OR Medication Therapy Management OR Medication Reconciliation OR Pharmacist Intervention OR clinical pharmacy

OR Management Medication Therapy OR Therapy Management Medication OR Drug Therapy Management OR Management Drug Therapy OR Therapy Management Drug OR Pharmaceutical Care OR Care Pharmaceutical OR Pharmacist Interventions OR Pharmacist Intervention OR Pharmacists Interventions OR Pharmacists Intervention OR medication management OR medication reconciliation OR Pharmaceutic Intervention OR Pharmaceutic Interventions OR

Pharmaceutical Interventions OR Pharmaceutical Intervention OR Pharmaceuticals Intervention OR Pharmaceuticals Interventions OR Clinical Pharmacists OR Clinical Pharmacist OR Pharmacist Clinical OR Pharmacists Clinical OR Pharmaceutical Service Community OR Pharmaceutical Services Community OR Service Community Pharmaceutical OR Services Community Pharmaceutical OR Pharmacy Services Community OR Community Pharmacy Service OR Pharmacy Service Community OR Services Community Pharmaceutic OR Services Community Pharmacy OR Community Pharmaceutic Services OR Community Pharmaceutic Service OR Pharmaceutic Service Community OR Pharmaceutic Services Community OR Service Community Pharmaceutic OR Community Pharmaceutical Services OR Community Pharmaceutical Service OR Service Community Pharmacy OR Pharmacy Service Clinical OR Service Clinical Pharmacy OR Clinical Pharmacy Services OR Pharmacy Services Clinical OR Services Clinical Pharmacy OR Clinical Pharmacy Service OR Pharmacy Residencies OR Pharmacy Residency OR Medication Management OR medication reconciliation

#3 COMPARADOR

Primary Health Care OR Community Health Services OR Preventive Medicine OR Secondary Care OR Comprehensive Health Care OR Community Care OR Secondary Health Care

OR Care Primary Health OR Primary Care OR Care Primary OR Primary Healthcare OR Healthcare Primary OR Health Services Community OR Community Health Service OR Health Service Community OR Service Community Health OR Services Community Health OR Community Health Care OR Care Community Health OR Health Care Community OR Community Healthcare OR Community Healthcares OR Healthcare Community OR Healthcares Community OR Community Health Services OR Preventative Medicine OR Medicine Preventative OR Preventive Medicine OR Preventive Care OR Care Preventive OR Preventative Care OR Care Preventative OR Health Care Comprehensive OR Comprehensive Healthcare OR Healthcare Comprehensive OR First Line Care OR Health Care Primary OR Community Care Service OR Community Care Services OR Preventive Health Care OR Secondary Health Care OR Secondary Healthcare OR Care Secondary OR Secondary Care OR Secondary Cares

Search strategy combined: 967
